# GRK6 palmitoylation dictates triple-negative breast cancer metastasis via recruiting the β-Arrestin 2/MAPKs/NF-κB signaling axis

**DOI:** 10.1186/s13058-024-01953-z

**Published:** 2024-12-31

**Authors:** Wen-Ke Wang, Hui-Yu Lin, Che-Hsuan Lin, Hsun-Hua Lee, Yen-Lin Chen, Yu-Hsien Kent Lin, Hui-Wen Chiu, Shry-Ming Sheen-Chen, Yuan-Feng Lin

**Affiliations:** 1 Graduate Institute of Clinical Medicine, College of Medicine, Taipei Medical University, Taipei, 11031 Taiwan; 2 Department of Surgery, Taipei Medical University Hospital, Taipei Medical University, Taipei, 11031 Taiwan; 3 Comprehensive Breast Center, Division of Breast Surgery and General Surgery, Department of Surgery, Cardinal Tien Hospital, Fu-Jen Catholic University, New Taipei City, Taiwan; 4 School of Medicine, Fu-Jen Catholic University, New Taipei City, Taiwan; 5 Department of Otolaryngology, Taipei Medical University Hospital, Taipei Medical University, Taipei, 11031 Taiwan; 6 Department of Otolaryngology, School of Medicine, College of Medicine, Taipei Medical University, Taipei, 11031 Taiwan; 7 Department of Neurology, Shuang Ho Hospital, Taipei Medical University, New Taipei City, 23561 Taiwan; 8 Department of Neurology, School of Medicine, College of Medicine, Taipei Medical University, Taipei, 11031 Taiwan; 9 Department of Neurology, Vertigo and Balance Impairment Center, Shuang Ho Hospital, Taipei Medical University, New Taipei City, 23561 Taiwan; 10 Department of Pathology, Tri-Service General Hospital, National Defense Medical Center, Taipei, Taiwan; 11 Department of Obstetrics and Gynaecology, North Shore Private Hospital, Sydney, NSW Australia; 12 Department of Gynecology, Ryde Hospital, Northern Sydney Local Health District, Sydney, Australia; 13 Northern Clinical School, Faculty of Medicine and Health, The University of Sydney, Sydney, NSW Australia; 14 Department of Medical Research, Shuang Ho Hospital, Taipei Medical University, New Taipei City, Taiwan; 15 TMU Research Center of Urology and Kidney, Taipei Medical University, Taipei, Taiwan; 16 Cell Physiology and Molecular Image Research Center, Wan Fang Hospital, Taipei Medical University, Taipei, Taiwan

**Keywords:** Triple-negative breast cancer, Metastasis, GRK6, β-Arrestin 2, MAPKs, NF-κB

## Abstract

**Background:**

Triple negative breast cancer (TNBC) belongs to the worst prognosis of breast cancer subtype probably because of distant metastasis to other organs, e.g. lungs. However, the mechanism underlying TNBC metastasis remains largely unknown.

**Methods:**

Bioinformatics analysis was conducted to evaluate the mRNA/protein expression and prognostic significance of G protein–coupled receptor kinase 6 (GRK6) in BC subtypes. RT-PCR assays were used to test the GRK6 expression in human BC tissues and cell lines. The in vitro cellular migration and in vivo lung colony-forming assays were established to estimate the metastatic potentials of TNBC cells. Western blotting was employed to examine protein phosphorylation, translocation and expression in the designed experiments.

**Results:**

Here we show that GRK6 upregulation is extensively detected in TNBC compared to normal mammary tissues and other BC subtypes and correlates with an increased risk for distant metastasis in TNBC patients. GRK6 knockdown suppressed but overexpression potentiated the cellular migration and lung colony-forming abilities of TNBC cells. Moreover, our data demonstrated that the posttranslational palmitoylation of GRK6 is extremely critical for activating β-Arrestin 2/mitogen-activated protein kinases (MAPKs)/NF-κB signaling axis and fostering the metastatic potentials of TNBC cells. Accordingly, the pharmaceutical inhibition of GRK6 kinase activity dramatically suppressed the activation of β-Arrestin 2, MAPKs and NF-κB and the cellular migration ability of highly metastatic MDA-MB231 cells. Sequentially blocking the β-Arrestin 2/MAPKs/NF-κB axis with their inhibitors predominantly mitigated the GRK6-promoted migration ability of poorly metastatic HCC1937 cells.

**Conclusion:**

Our results not only provide a novel mechanism for TNBC metastasis but also offer a new therapeutic strategy to combat metastatic TNBC via targeting GRK6 activity.

**Supplementary Information:**

The online version contains supplementary material available at 10.1186/s13058-024-01953-z.

## Background

Breast cancer is the most common cancer, about 30% of all new cancer, diagnosed in women worldwide [1]. The clinical medications were developed according to the classification of breast cancer subsets [2]. Triple-negative breast cancers (TNBCs) are defined as their negative expression for the estrogen receptor (ER), progesterone receptor (PR) and human epidermal growth factor receptor 2 (HER2). With the advance in genomics, breast cancer has further classified into 5 molecular subtypes, including luminal A, luminal B, HER2-enriched, normal-like and basal-like breast cancer, with different prognostic value [3]. It has been shown that about 75% of basal like breast cancer are TNBC. Unlike ER-positive and HER2-enriched breast cancers have effective hormonal (e.g., tamoxifen) and targeted (e.g., Herceptin) therapies, respectively, the conventional chemotherapy is still a main regiment for treating TNBCs. However there is only a subgroup of TNBCs showing a favorable prognosis [4]. As a result, drug resistance and distant metastasis become major clinical issues in the management of TNBCs and the main causes of poorest prognosis for TNBCs among BC subtypes.

G protein–coupled receptors (GPCRs) are consisted with the largest family of cell surface receptors and control a broad spectrum of biological functions [5]. GPCR kinases (GRKs) rapidly phosphorylate the agonist-bound GPCRs and subsequently recruit β-Arrestins to interact with the C-terminal of phosphorylated GPCRs. β-Arrestins have multifunction, such as adaptors, scaffolds and signal transducers, and connect the activated GPCRs with diverse signaling pathways, e.g. MAPKs and NF-κB, within the cell [6]. The GRK family comprise 7 mammalian members, among which GRK2, GRK3, GRK5, and GRK6 are expressed ubiquitously [7]. Different GRKs has been shown to phosphorylate distinct sites on the C terminus of the targeted receptors, which establishes a barcode that determines the functional consequences of β-Arrestin engagement [8]. GRK6-mediated GPCR phosphorylation and activation β-Arrestins has been shown to play an important role in regulating lymphocyte migration and immune responses [9]. In colorectal carcinoma, GRK6 upregulation was appeared to associate with the metastatic progression and poor outcomes in cancer patients [10]. Similarly, overexpression of GRK6 was associated with an increased risk for cancer metastasis and a poorer prognosis in papillary thyroid carcinoma [11] and hepatocellular carcinoma [12]. Conversely, the suppression of GRK6 by the activated growth factor receptor-src axis was found to promote medulloblastoma migration [13]. Using a murine models of human lung cancer, it has been reported that GRK6 deficiency promotes angiogenesis, tumor progression and metastasis [14]. In human lung adenocarcinoma, the hypermethylation of GRK6 promoter, the knockdown of GRK6 gene or a decreased expression of GRK6 protein was shown to correlate with an enhanced metastatic potentials and poorer prognosis [15, 16]. These findings indicate a controversial prognostic significance of GRK6 in different types of cancer. In breast cancer, the prognostic significance of GRK6 or its role in modulating cancer metastasis remains largely unknown even though GRK2 and GRK4, not GRK3, upregulation have been found to promote tumorigenesis, migration/invasion and metastasis in breast cancer cells [17–19].

As a result, the efforts of this study were aimed to explore the functional roles of GRK6 in promoting the metastatic progression of TNBC and its related signaling pathways. We found that GRK6 upregulation is extensively detected in TNBC as compared to non-TNBC and normal tissues and refers to a poorer recurrence/distant metastasis-free survival probability in TNBC patients. Whereas GRK6 knockdown dramatically suppressed the cellular migration ability and lung metastatic colony-forming capability of highly metastatic TNBC cell line MDA-MB231, GRK6 overexpression predominantly enhanced the metastatic potentials of poorly metastatic HCC1937 cells. We found that the palmitoylated GRK6 recruits β-Arrestin 2 to activate MAPK/NF-κB signalling axis and thereby ultimately promote the metastatic progression of TNBC. Our findings provide a novel mechanism for TNBC metastasis and a new strategy to combat metastatic TNBC via targeting GRK6 activity.

## Methods

### Transcriptional profiles and clinical tissues of breast cancer patients

The results of mRNA expression determined by RNAseq (polyA þ Illumina HiSeq) analysis deposited in the TCGA breast cancer database were also downloaded from the UCSC Xena website (UCSC Xena. Available online: http://xena.ucsc.edu/welcome-to-ucsc-xena/, accessed on 1 January 2022). Kaplan-Meier Plotter was employed to estimate the prognostic significance of GRK6 in breast cancer subtypes. The patients were stratified into low and high GRK expression groups under a maximal risk condition (minimized *p* value) in Kaplan-Meier analyses. The Hallmark gene sets used in this study were downloaded from Molecular Signature Database (https://www.gsea-msigdb.org/gsea/msigdb, accessed on 1 January 2022). Breast cancer tissues were obtained from Cardinal Tien Hospital and collected in accordance with institutional review board approval (CTH-101-3-5-054) and the Declaration of Helsinki.

### Cell culture conditions

TNBC cell lines were obtained from American Type Culture Collection (ATCC) and cultivated in Leibovitz’s (L-15) medium (for MDA-MB231) and RPMI-1640 medium (for HCC38, HCC1806, and HCC1937) supplemented with 10% fetal bovine serum (FBS). Except MDA-MB231 cells incubated at 37 °C in a free gas exchange with atmospheric air, other cell lines were cultured at 37 °C with 5% CO_2_. All cell lines are obtained from American Type Culture Collection (ATCC). All media and supplements were purchased from Gibco Life Technologies (Thermo Fisher Scientific Inc., Waltham, MA, USA).

### Migration assay

Cellular migration assay was performed in the trans-well culture using Boyden chamber system (Neuro Probe, Inc., Gaithersburg, MD, USA). Briefly, the lower chamber was fulfilled the conditioned media prior to covering with a polycarbonate membrane (8 μm pore size, Neuro Probe, Inc.) pre-coated with 10 µg of human fibronectin on the immersed site (Sigma, St. Louis, MO, USA). Cells (1.5 × 10^4^) were seeded into the top chamber containing 50 µL of serum-free medium. After 4 h, the remaining stationary cells on the top side of the membrane were removed prior to fixing the migrated cells on the bottom side of the membrane with 100% methanol followed by performing the staining with 10% Giemsa’s solution (Merck, Levelingstr, Munchen, Germany) for 1 h. Migrated cells were counted under the light microscope from three independent experiments.

### Cloning and lentiviral preparation/infection

Human cDNA clones for GRK6A (NM_001004106.3) and GRK6B (NM_002082) were obtained from the National RNAi Core Facility Platform in Taiwan and Sino Biological Inc. (Beijing, China), respectively, and subcloned into lentivirus shuttle vector pLAS/3w with puromycin-resistant gene. Lentiviral particles with vector only or vector-containing GRK6 gene variants were produced through the collaboration with the National RNAi Core Facility. Lentiviral particles containing a puromycin-resistant gene and non-silencing control or GRK6 shRNAs [sh1 (TRCN0000001367): CCGG*CCTCGACAGCATCTACTTCAA*CTCGAGTTGAAGTAGATGCTGTCGAGGTTTTT; sh2 (TRCN0000010618): CCGG*GTGTTAGGGTAGCATGGGATT*CTCGAGAATCCCATGCTACCCTA ACACTTTTT] were purchased from the National RNAi Core Facility. Cells with a density at 50% confluence grown in 6-well plates were replenished with the fresh conditioned media containing 5 µg/ml of polybrene (Santa Cruz, Dallas, TX, USA) prior to performing the infection with the produced lentiviral viral particles at 2–10 multiplicity of infection (MOI) overnight. The vector control/GRK6-overexpressing HCC1937 cells and the non-silenced control/GRK6-silenced MDA-MB231 cells were obtained after the cultivation in the presence of puromycin (10 µg/ml) for 24 h and then subjected to RT-PCR and Western blot analyses for confirming the efficiency of gene overexpression and knockdown.

### Reverse transcription PCR (RT-PCR)

Total RNA of primary tumors and normal adjacent tissues from clinical breast cancer patients and detected TNBC cells was extracted by using TRIzol extraction kit (Invitrogen). The aliquots of total RNA (5 µg) were incubated with M-MLV reverse transcriptase (Invitrogen) to yield cDNA products which were subsequently amplified by PCR protocol with a Taq-polymerase (Protech, Taipei, Taiwan) using paired primers (for GRK6, forward-CAGAGGAAGAAGAAGATCAAGCGG and reverse-GACATTCAGCTCTTGGAAGCACTC for GAPDH, forward-AGGTCGGAGTCAAC GGATTTG and reverse-GTGATGGCATGGACTGTGGTC).

### Western blot analysis

Whole cell lysates (30–100 µg) and membrane (300 µg)/cytosolic (100 µg) fractions that were extracted by a commercial kit (Thermo Fisher Scientific Inc.) resuspended in the loading buffer [62.5 mM Tris (pH 6.7), 1.25% SDS, 12.5% glycerol, and 2.5% β-mercaptoethanol] were boiled for 5 min prior to performing SDS-PAGE experiment. After transferred to PVDF membrane, the membranes were incubated with blocking buffer (5% bovine serum albumin for detecting phosphorylated proteins or 5% skim milk for detecting other protein in TBS containing 0.1% Tween-20) for 1 h at room temperature. Subsequently, the proteins on the membrane were incubated with antibodies against GRK6 from CUSABIO (Houston, TX, USA), phosphorylated β-Arrestin (p-β-Arrestin), β-Arrestin, p-p38, p38, p-Erk1/2, Erk1/2, p-NF-κB (Ser536, NF-κB, E-cadherin, N-Cadherin, Vimentin and Slug from Cell Signaling (Danvers, MA, USA), Fibronectin from Abcam (Cambridge. UK), and GAPDH from AbFrontier (Seoul, Korea) overnight at 4 °C. After several washes, the membranes were further incubated with a peroxidase-labeled secondary antibody for another 1 h at room temperature. Immunoreactive bands were visualized using an enhanced chemiluminescence system (Amersham Bioscience, Tokyo, Japan). The membranes for detecting phosphorylated proteins were re-probed with antibodies against the respective total protein (Uncut blots are shown in the Fig. S9–S15).

### Luciferase-based reporter assay

Luciferase reporter vectors containing NF-κB response element were purchased from Promega (Madison, WI, USA.) Cells with a density at 70% confluence grown on in 6-well plates were co-transfected with luciferase reporter and Renilla luciferase expression vectors. Post-transfection for 24 h, Dual-Glo^®^ Luciferase Assay System purchase from Promega was used to detect the cellular luciferase activities according to the manufactural guideline. The level of firefly luminescence was finally normalized to that of Renilla luminescence.

### Immunohistochemistry staining

Tissue microarray (TMA) containing normal adjacent tissues and primary tumors of breast cancer patients and the related clinical information was obtained from Superbiochips (Cat. CBA6). Deparaffinized TMA tissues were subjected to antigen retrieval using 10 mM citrate buffer (pH = 6.0) and then incubated with 3% H2O2 solution to eliminate endogenous peroxidase activity. The TMA tissues were further incubated with normal goat serum (1:20) for 1 h to prevent the non-specific immunoreactions prior to incubate with GRK6 antibody (CSB-PA009927ESR1HU, 1:500) in a humidified chamber at 4℃ for overnight. After excess washes, the TMA tissues were subsequently incubated with peroxidase-conjugated secondary antibody (N- Histofine^®^ Simple Stain Mouse MAX PO, Comso Bio) at 100 µl for another 1 h. Finally, the immunoreactive proteins were visualized by incubating with DAB reagent (PolyDetector Liquid DAB HRP Brown Kit). Counterstaining was performed with Hematoxylin. Slides were dehydrated, cleared in xylene, mounted, and observed under a microscope.

### Gene set enrichment analysis

The fold change of mRNA levels derived from the comparison of RNA-sequencing results between the GRK6-knockdown and non-silencing control ATII cells in GSE164921 was generated by using GEO2R program. The ranked somatic genes by fold change were subjected to the in silico experiment using Gene Set Enrichment Score (GSEA) program against BIOCARTA gene sets. The Spearman’s correlation test was used to analyze the GRK6 co-expression with other somatic genes in the TNBC samples from The Cancer Genome Atlas database. The ranked somatic genes by Spearman’s rho value were then analyzed by GSEA program against HALLMARK gene sets.

### Animal studies

Metastatic lung colony-forming assays were established by transplanting HCC1937 and MDA-MB231 cell variants (1 × 10^6^ cells resuspended in 100 µL PBS) into Advanced-Severe-ImmunoDeficiency (ASID) B6.129S4-Il2rg^tm1Wjl/J^ mice obtained from the National Laboratory Animal Center in Taiwan through the tail-vein injection. All procedures of animal experiment were reviewed and approved (LAC-2019-0060) by the Institutional Animal Care and Use Committee at Taipei Medical University. Post-transplantation for 4 weeks, mice were humanely killed and lungs were obtained for histological analysis.

### Statistical analysis

SPSS 17.0 software (Informer Technologies, Roseau, Dominica) was used to analyze statistical significance. Paired t-test was utilized to compare GRK6 gene expression in the cancer tissues and corresponding normal tissues. Evaluation of survival probabilities were determined by Kaplan-Meier analysis and log-rank test. One-way ANOVA using Tukey’s post hoc test was used to analyze the statistical significance of the detected gene expression in clinical samples. The Non-parametric Mann-Whitney U test was used to analyze the data from cell-based and animal experiments. In all statistical analyses, *p* values < 0.05 was considered statistically significant.

## Results

### GRK6 upregulation is extensively found in TNBC compared to non-TNBC and normal tissues and significantly correlates with a higher risk for distant metastasis in TNBC patients

**Fig. 1:**
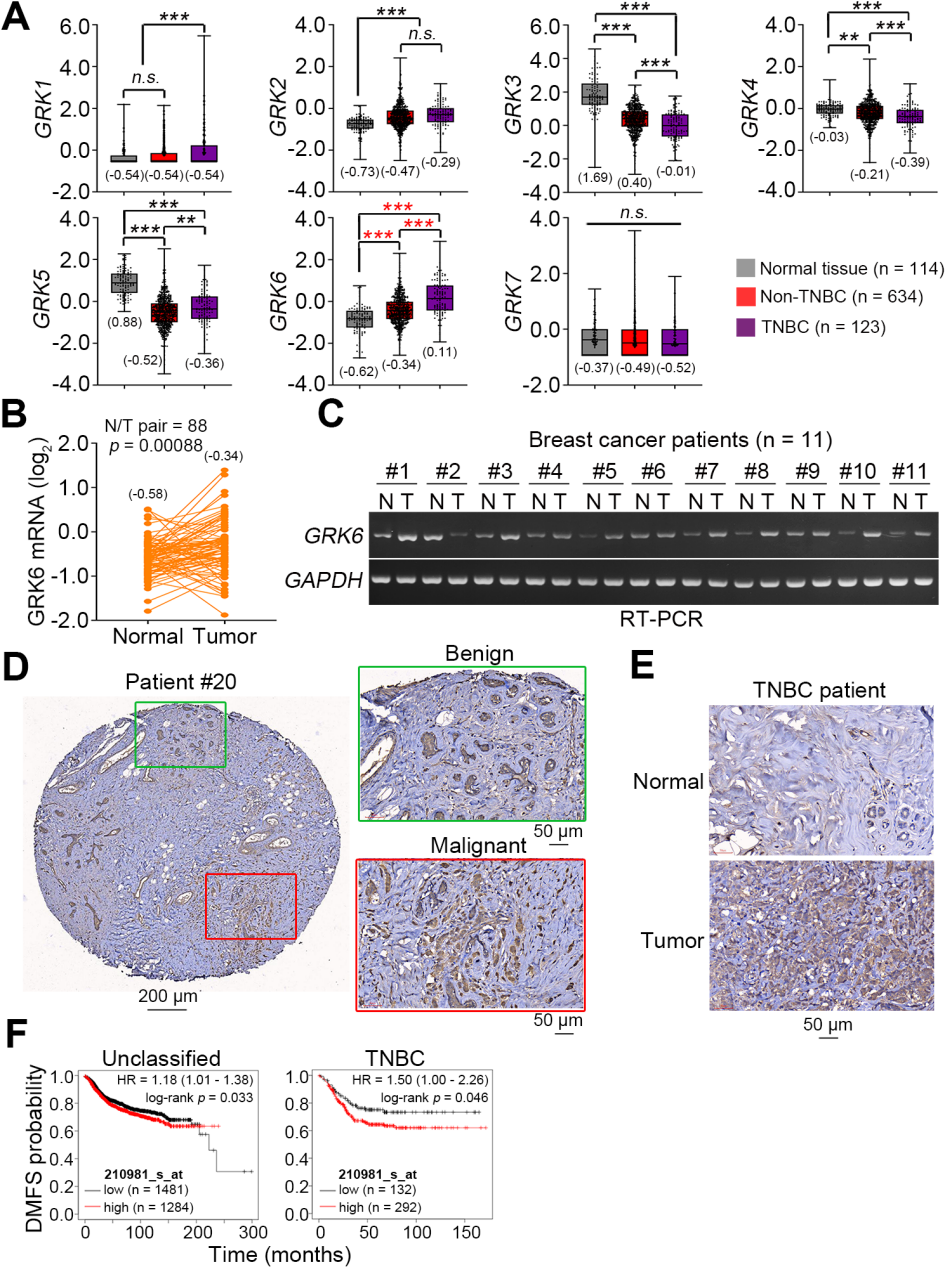
Transcriptional profiling of GRK6 in breast cancer. **A** Boxplot for the mRNA levels of GRK1-7 in TCGA normal mammary epithelial tissues, primary tumors derived from non-TNBC or TNBC. In A, the symbols “**” and “***” indicate statistical differences at *p* < 0.01 and *p* < 0.001, respectively, analysed by One-way ANOVA using Tukey’s post hoc test. **B** Points with connecting lines plot for GRK6 transcriptional levels detected in adjacent normal tissues and primary tumors from TCGA breast cancer database. Statistical significance was analysed by paired t-test. In B, the inserts represent the average of GRK6 mRNA levels. **C** RT-PCR experiments for the transcriptional levels of GRK6 and GAPDH in the paired normal tissues (N) and primary tumors (T) derived from 11 breast cancer patients from Cardinal Tien Hospital. GAPDH was used as an internal control of RT-PCR experiments. **D**–**E** Immunohistochemistry staining for GRK6 protein against the benign (green)/malignant (red) regions (D) and paired normal tissue/primary tumors (E) derived from TNBC patients. **F** Kaplan-Meier analyses for GRK6 gene (probe ID: 210981_s_at) expression under a distant metastasis-free survival (DMFS) probability using maximal risk condition (at a minimal *p* value) against the unclassified and TNBC patients from K-M Plotter Website. The abbreviation of HR in the insert denotes hazard ratio

First of all, we dissected the transcriptional profiling of GRK subfamily members (GRK1-7) in primary tumors and normal mammary epithelial tissues from TCGA breast cancer database. We found that GRK6 gene expression in primary tumors derived TNBC patients is significantly (*p* < 0.001) higher than that of normal tissues and primary tumors derived non-TNBC patients in TCGA breast cancer database (Fig. 1A). Moreover, in the paired normal adjacent tissues and primary tumors, the transcriptional levels of GRK6 was detected to be relatively higher in primary tumors compared to adjacent normal tissues (Fig. 1B). Accordingly, RT-PCR results showed that GRK6 upregulation are commonly found in primary tumors compared to adjacent normal tissues derived from 11 breast cancer patients (Fig. 1C). Immunohistochemistry (IHC) staining results derived from The Human Protein Atlas database revealed that GRK6 protein expression in breast cancer tissues is higher than that in normal mammary tissues and mainly distributed in cytoplasm and cell membrane (Fig. S1A). We further validated these findings by performing IHC staining for GRK6 protein levels against the commercial tissue microarray of breast cancer. The data showed that malignant breast turmor compared to benign mammary tissues harbour a higher GRK6 expression (Fig. 1D). The IHC staining also revealed that GRK6 is upregulated in the primary tumors compared to adjacent normal tissues from TNBC patients (Fig. 1E). Kaplan-Meier analyses demonstrated that a higher level of GRK6 transcript is closely associated with a poorer distant metastasis-free survival probability in TNBC patients compared to patients with unclassified breast cancers s (Fig. 1F). Accordingly, IHC staining results indicated that GRK6 upregulation and membrane translocation probably associates with lymph node metastasis of breast cancer (Fig. S1B and C) These findings implicate that GRK6 may act a pivotal role in the molecular mechanism underlying the metastatic progression of TNBC.

### GRK6 expression causally associates with the metastatic potentials of TNBC cells in vitro and in vivo

**Fig. 2:**
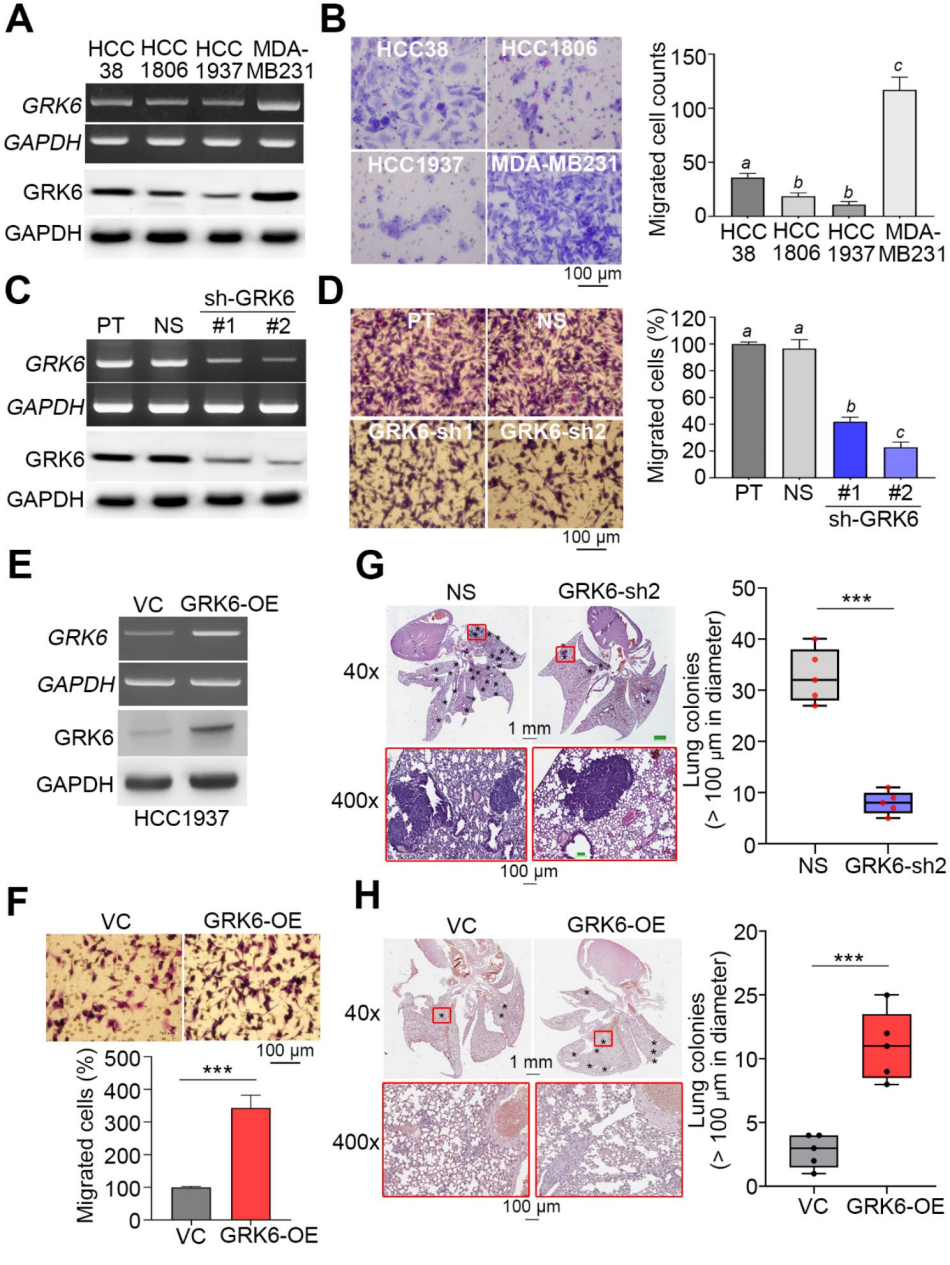
GRK6 expression associates with the metastatic potentials of TNBC cells. **A** RT-PCR (upper) and Western blot (lower) analyses for the mRNA and protein levels of GRK6 and GAPDH in the detected TNBC cell lines. **B** Giemsa stain (left) for the migrated cells in a trans-well cell culture for the detected TNBC cell lines and histogram (right) for the migrated cell number from three independent experiments. **C** RT-PCR (upper) and Western blot (lower) analyses for the mRNA and protein levels of GRK6 and GAPDH in MDA-MB231 cells stably transfected without (parental, PT) or with non-silencing (NS) control or 2 independent GRK6 shRNA clones. **D** Giemsa stain (left) for the migrated cells in a trans-well cell culture for the MDA-MB231 cell variants and histogram (right) for the migrated cell number from three independent experiments. **E** RT-PCR (upper) and Western blot (lower) analyses for the mRNA and protein levels of GRK6 and GAPDH in HCC1937 cells without (vector control, VC) or with GRK6 overexpression (OE). **F** Giemsa stain (left) for the migrated cells in a trans-well cell culture for the HCC1937 cell variants and histogram (right) for the migrated cell number from three independent experiments. **G** Haematoxylin/eosin (H&E) staining (left) and box plot (right) for lung colonies of MDA-MB231 cell variants from tumor-bearing mice (*n* = 6). **H** H&E staining (left) and box plot (right) for lung colonies of HCC1937 cell variants from tumor-bearing mice (*n* = 6). GAPDH in A, C and F was used as an internal control of experiments. In B and D, the different alphabets indicate the statistical significance at *p* < 0.05. In F, G and H, the symbol “***” denotes the statistical significance at *p* < 0.001

To ascertain if GRK6 expression is associated with metastatic potentials of TNBC, we next performed a cellular migration assay using a trans-well cell culture against TNBC cell lines HCC38, HCC1806, HCC1937 and MDA-MB231. Whereas HCC1806 and HCC1937 cells harboring a lower expression of endogenous GRK6 expression exhibited a poorer migration ability, HCC38 and MDA-MB231 cells with a higher endogenous GRK6 levels showed a relatively stronger migration ability in a trans-well culture for 16 h (Fig. 2A and B). Similar to clinical samples, TNBC cell lines HCC38, HCC1806, HCC1937, Hs578T., MDA-MB231, MDA-MB468 and MDA-MB453 exhibited a relatively higher levels of GRK6 as compared to non-tumor mammary cell lines H1845F5 (H184) and MCF10A and ER + cell lines MCF7, T47D and BT-20 (Fig S2). The knockdown of GRK6 gene using its 2 independent shRNA clones in MDA-MB231 cells predominantly repressed the endogenous levels of GRK6 (Fig. 2C) and significantly (*p* < 0.001) suppressed the cellular migration ability (Fig. 2D). Conversely, the enforced expression of exogenous GRK6A gene (Fig. 2E) dramatically enhanced the migration (Fig. 2F). Robustly, GRK6 knockdown mitigated the lung colony-forming ability of MDA-MB231 cells in comparison with the non-silencing control cells in the tumor-bearing mice (Fig. 2G). In contrast, GRK6 overexpression as compared to vector control dramatically enhanced the lung colony-forming abilities of HCC1937 cells (Fig. 2H and Fig. S3).

### GRK6 membrane association activity is required for promoting the process of epithelial-mesenchymal transition and cellular migration ability in TNBC cells

**Fig. 3:**
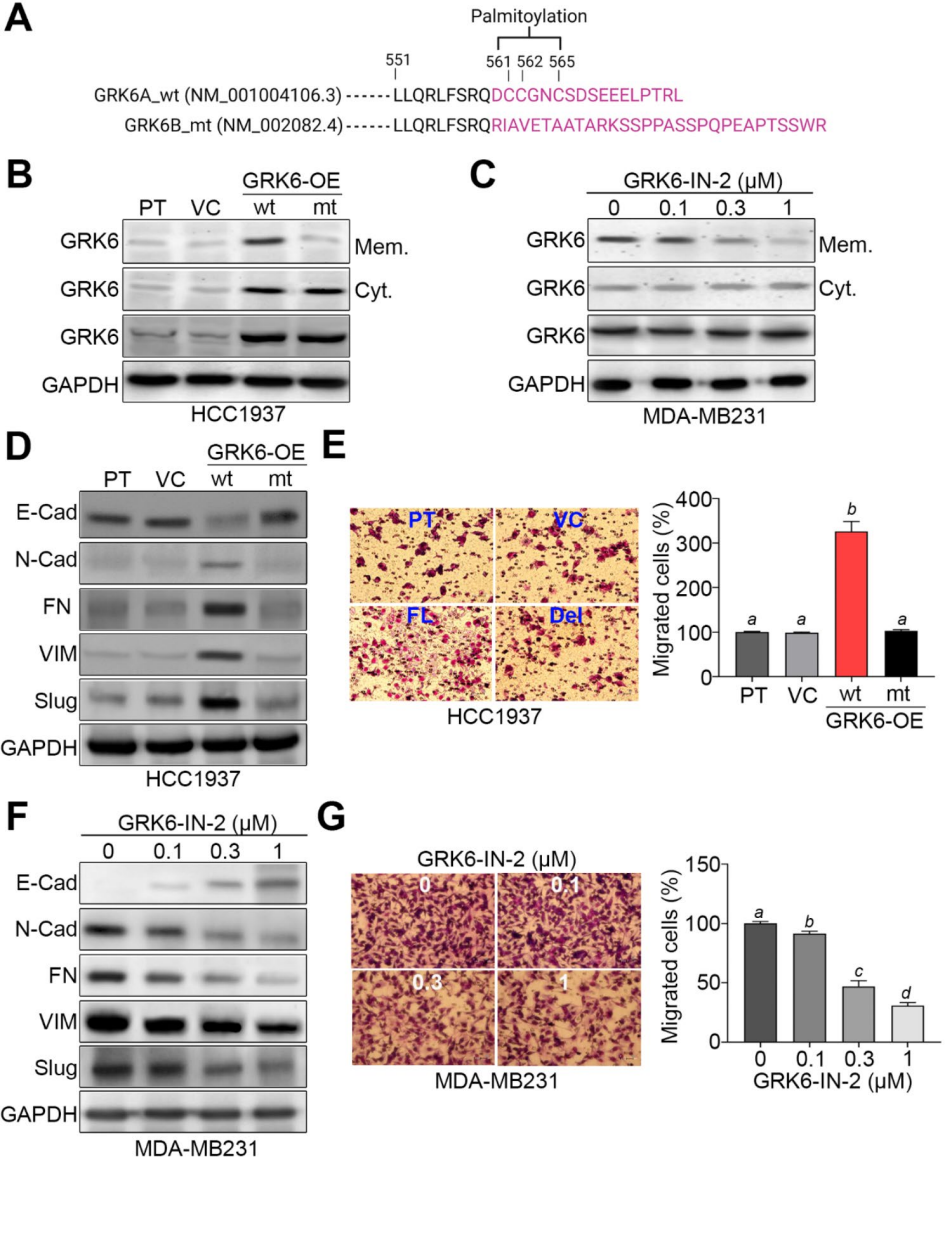
GRK6 palmitoylation and kinase activity determine the EMT progression and cellular migration activity in TNBC cells. **A.** The illustration for the difference of amino acid sequence (highlighted at pink) at the C-terminal region of palmitoylatable GRK6A (wild-type, wt) and non-palmitoylatable GRK6B (mutant, mt) variants. **B–C.** Western blot analyses for protein levels of membrane (Mem.)-associated/cytosolic (Cyt.)/total GRK6 and GAPDH in the parental (PT) HCC1937 cells without or with overexpression of vector control, GRK6A or GRK6B (B) and MDA-MB231 cells treated with GRK6 inhibitor GRK6-IN-2 at the indicated concentrations for 24 h (C). **D–E.** Western blot analyses (D) for GAPDH and EMT markers E-cadherin (E-Cad), N-cadherin (N-Cad), Fibronectin (FN), Vimentin (VIM) and Slug in the whole cell lysates, and Giemsa stain (E, left) for the migrated cells in the trans-well cultivation of HCC1937 cell variants. The migrated cell number from three independent experiments were presented as mean ± SEM in the histogram (E, right). **F–G.** Western blot analyses (F) for GAPDH and the indicated EMT markers in the whole cell lysates, and Giemsa stain (G, left) for the migrated cells in the trans-well cultivation of MDA-MB231 cells treated with GRK6-IN-2 at the indicated concentrations for 24 h. The migrated cell number from three independent experiments were presented as mean ± SEM in the histogram (G, right). In B, C, D and F, GAPDH was used as an internal control of protein loading. In E and G, the different alphabets indicate the statistical significance at *p* < 0.05

Since the C-terminal palmitoylation of GRK6 is a critical process for its membrane translocation and subsequent interaction with an agonist-activated receptor [20], we generated HCC1937 cells overexpressing 2 exogenous GRK6 gene variants GRK6A with the palmitoylation sites at Cys-561/562/565 and GRK6B lacking the palmitoylation sites at C-terminal region (Fig. 3A). Thereafter, we defined GRK6A gene as wild-type and GRK6B as mutant form in this study. Western blot analyses indicated that the overexpression of wild-type, not mutant, GRK6 gene trigger the membrane localization of GRK6 in HCC1937 cells (Fig. 3B). The similar views were also found in the HCC1937 cells transfected with GRK6 gene with the mutations at the palmitoylation sites (Fig. S4A and B). Accordingly, the restoration of GKR6A, not GRK6B, dramatically rescued the migration ability of GRK6-silenced MDA-MB231 cells (Fig. S4C and D), indicating the driver effect of GRK6 on TNBC metastatic progression. Besides, the pharmaceutical inhibitor of GRK6 kinase activity by GRK-IN-2 dose-dependently suppressed the membrane localization of GRK6 in highly metastatic MDA-MB231 cells (Fig. 3C). The data from Gene Set Enrichment Analysis (GSEA) demonstrated that GRK6 upregulation in TNBC probably associates with the progression of epithelial-mesenchymal transition (Fig. S5A and C). Furthermore, our data showed that wild-type, not mutant, GRK6 overexpression triggers EMT progression as judged by the decreased level of E-type marker E-cadherin and increased levels of M-type markers N-cadherin, Fibronectin, Vimentin and Slug (Fig. 3D). Accordingly, the lack of palmotoylation sites in the C-terminal region of GRK6 protein failed to force the cellular migration ability in HCC1937 cells (Fig. 3E). On the other hand, the treatment with GRK6 kinase inhibitor GRK6-IN-2 dose-dependently suppressed the EMT progression (Fig. 3F) and cellular migration ability (Fig. 3G) in the highly metastatic MDA-MB231 cells.

### GRK6 recruits β-Arrestin 2 to promote the metastatic progression in TNBC cells

**Fig. 4:**
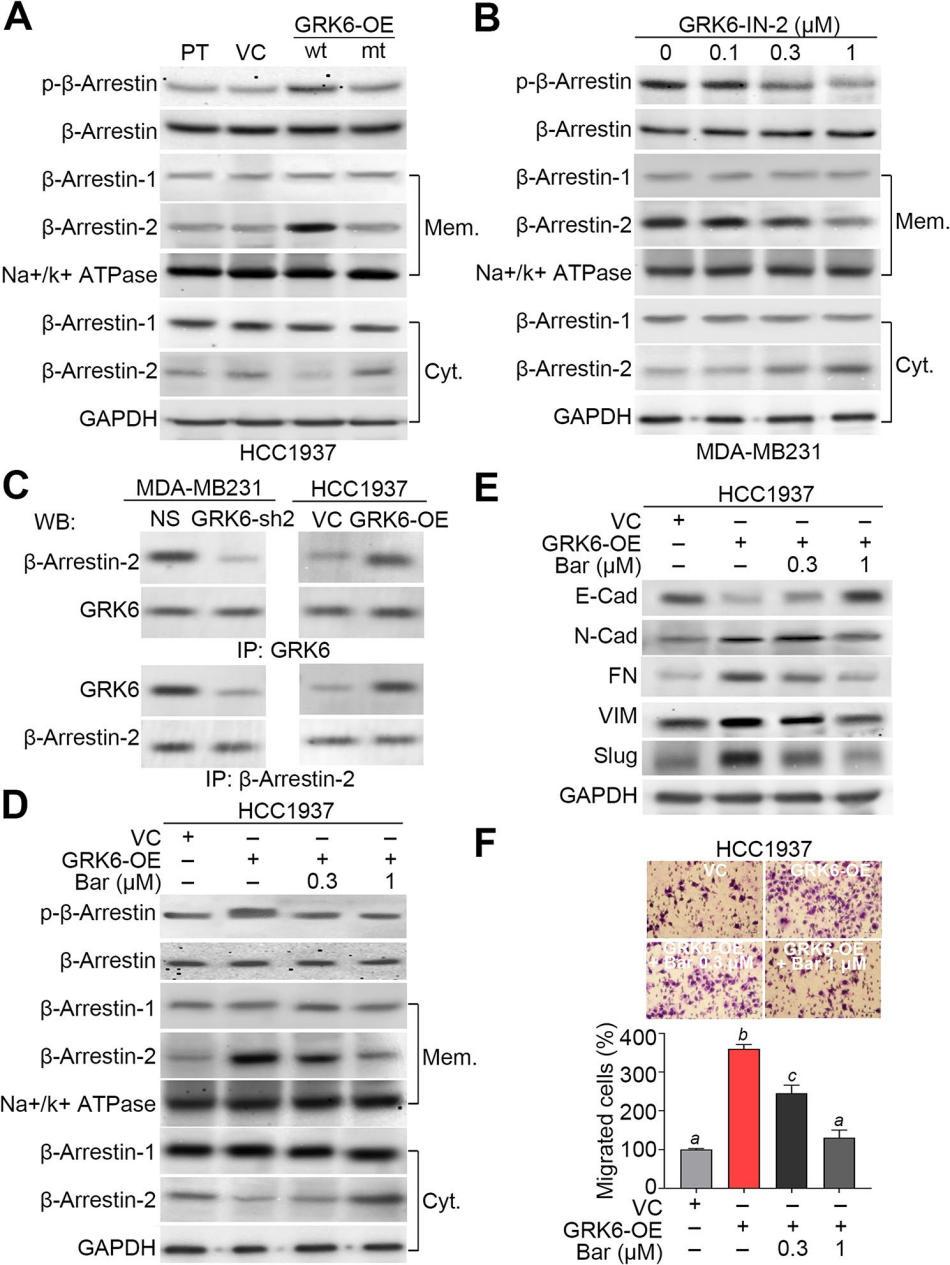
GRK6 palmitoylation and kinase activity recruit the membrane localization of β-Arrestin to trigger the EMT progression and cellular migration activity in TNBC cells. **A**–**B**. Western blot analyses for the phosphorylated β-Arrestin (p-β-Arrestin), total β-Arrestin, membrane (Mem.)-associated/cytosolic (Cyt.) β-Arrestin 1/2, Na+/K+ATPase and GAPDH in the parental (PT) HCC1937 cells without or with overexpression of vector control (VC), GRK6A or GRK6B (A) and in MDA-MB231 treated with GRK6 inhibitor at the indicated concentrations for 24 h (B). **C**. Immunoprecipitation assay for GRK6:β-Arrestin complex in the NS/GRK6-sh2 MDA-MD231 cells and VC/GRK6-OE HCC1937 cells. **D**–**F**. Western blot analyses for β-Arrestin 1/2 (D) and the EMT markers (E) in the whole cell lysate and Giemsa stain for the migrated cells in the trans-well cultivation (F, upper) of vector control HCC1937 cells and GRK6A-overexpressing HCC1937 cells treated without or with β-Arrestin inhibitor at the indicated concentrations for 24 h. The migration cell number from three independent experiments were presented as mean ± SEM in the histogram (F, lower). GAPDH in A, B, D and E was used as an internal control of total and cytosolic protein loading. Na+/K+ATPase in A, B and C was used as an internal control of membrane protein loading. In E, the different alphabets indicate the statistical significance at *p* < 0.05

Because the interaction of GRK6 with β-Arrestin was found to regulate several cellular functions, we next examined the requirement of β-Arrestin activity for the GRK6-promoted metastatic progression in TNBC. Western blot analyses demonstrated that the enforced expression of wild-type, not mutant, GRK6 gene induces the membrane translocation and phosphorylation of β-Arrestin in HCC1937 cells (Fig. 4A). To delineate which β-Arrestin subtype is responsible for GRK6 membrane localization and activation, we next performed the membrane/cytosolic protein extraction. Another Western blot analysis revealed that β-Arrestin 2, not β-Arrestin 1, is activated and recruited to cell membrane in the GRK6A, not GRK6B, -overexpressing HCC1937 cells (Fig. 4A). The pharmaceutical inhibition of GRK6 kinase activity by GRK6-IN-2 predominantly suppressed phosphorylation of β-Arrestin and the membrane translocation β-Arrestin 2, not β-Arrestin 1, in MDA-MB231 cells in a dose-dependent manner (Fig. 4B). Whereas GRK6 knockdown in MDA-MB231 cells decreased the complex of GRK6: β-Arrestin 2, GRK6 overexpression in HCC1937 cells promoted the formation of GRK6: β-Arrestin 2 (Fig. 4C). To delineate if the activity of β-Arrestin is exactly needed for the GRK6-triggered metastatic progression in TNBC, we next employed a selective β-Arrestin inhibitor Barbadin which is capable of blocking the agonist-promoted endocytosis of its associated receptors [21]. The treatment with Barbadin dramatically inhibited the GRK6-enhanced β-Arrestin phosphorylation/β-Arrestin 2 membrane translocation (Fig. 4D), EMT progression (Fig. 4E) and cellular migration ability (Fig. 4F) in the GRK6A-overexpressing HCC1937 cells. Similar views were also found in the highly metastatic MDA-MB231 cells (Figure S6A – C).

### GRK6/β-Arrestin 2-enhanced metastatic potentials of TNBC cells is mediated by the MAPK-NF-κB signalling axis

**Fig. 5:**
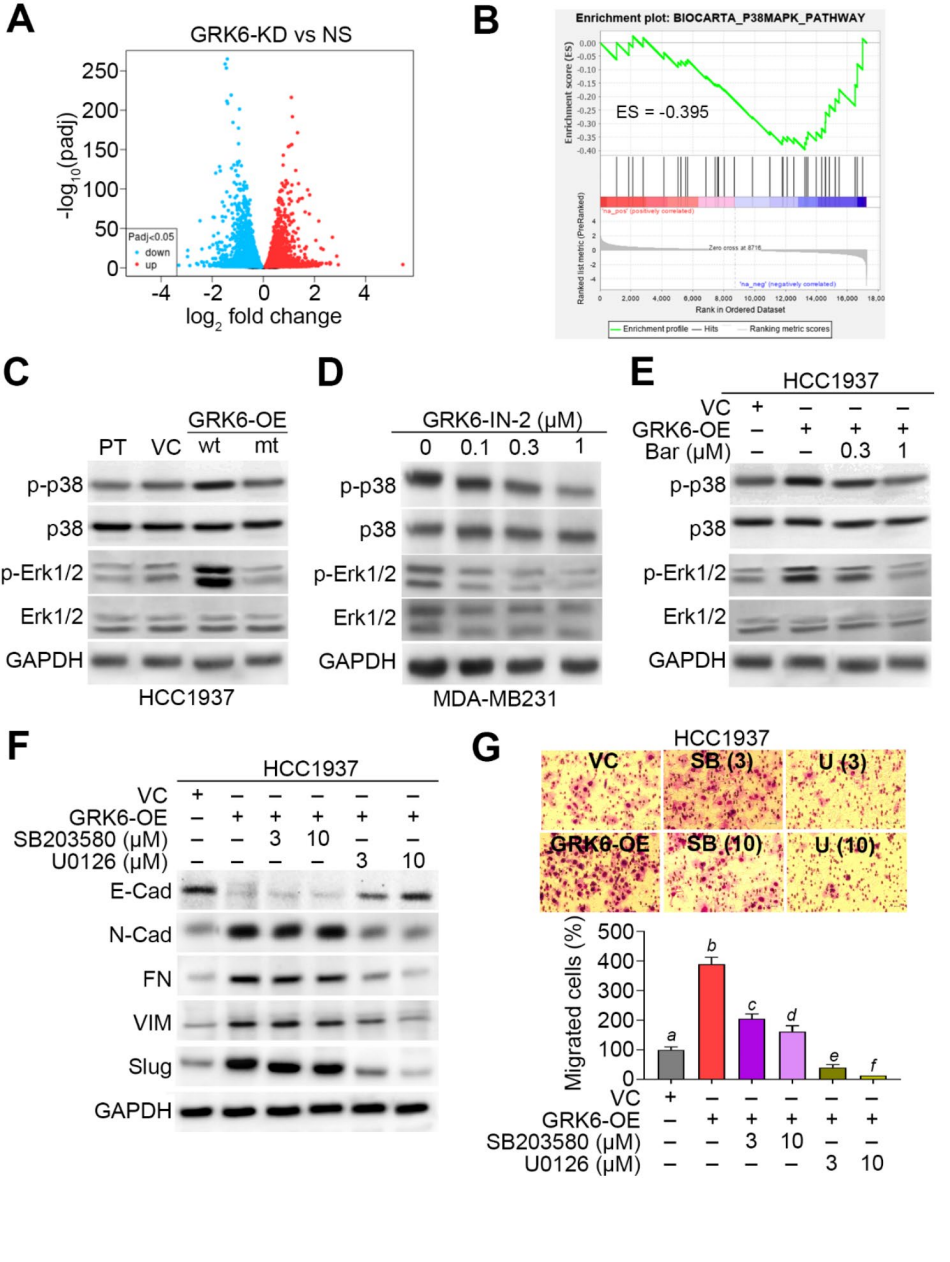
GRK6 and β-Arrestin activation promote the EMT progression and cellular migration activity by activating MAPKs in TNBC cells. **A** Volcano plot for log_2_ fold change and –log_10_ [adjusted *p* value (padj)] derived from the upregulated (red) and downregulated (blue) genes in the GRK6-silenced cells as compared to the non-silencing control cells in GSE164921 dataset. **B** The plot of enrichment score (ES) for the correlation between P38MAPK_PATHWAY gene set and the ranked somatic genes by log_2_ fold change shown in A. **C–E.** Western blot analyses for the phosphorylated p38 (p-p38), total p38, p-Erk1/2, total Erk1/2 and GAPDH in whole cell lysates from the indicated HCC1937 cell variants (C), MDA-MB231 cells treated without or with GRK6 inhibitor at the indicated concentrations (D), and the VC/GRK6A-OE HCC1937 cells treated without or with β-Arrestin inhibitor at the indicated concentrations (E). **F–G**. Western blot analyses for the EMT markers in the whole cell lysates (F), and Giemsa stain for the migrated cells in the trans-well cultivation (G, upper) of VC and GRK6A-OE HCC1937 cells treated without or with p38 inhibitor SB203580 and Erk1/2 inhibitor U0126 at the indicated concentrations for 24 h. The migration cell number from three independent experiments were presented as mean ± SEM in the histogram (G, lower). The different alphabets indicate the statistical significance at *p* < 0.05. In C, D, E and F, GAPDH was used as an internal control of protein loading

The interaction of GRK/β-Arrestin was found to foster cell migration via regulating the mitogen-activated protein kinase (MAPK) pathway [22]. To validate this scenario, we re-analyzed the RNA-sequencing results against the non-silencing control and GRK6-silenced ATII cells from GSE164921 data set. In comparison with the non-silencing control ATII cells, GRK6 knockdown robustly repressed its mRNA levels in 3 independent samples (Fig. S7A and B) and significantly (*p* < 0.001) displayed a great fold-change of mRNA expression in the downregulated gene cluster (Fig. 5A and Fig. S1C). Furthermore, the in silico experiment using GSEA program revealed that the ranked somatic genes by the fold change of mRNA expression as shown in Fig. 5A negatively correlates with the gene set of P38MAPK_PATHWAY (Fig. 5B.), indicating a regulatory role of GRK6 towards MAPK activation. Based on this finding, we next investigate the requirement of MAPK for the GRK6-enhanced metastatic potentials of TNBC. We found that the enforced expression of wild-type, not mutant, GRK6 induces the protein levels of phosphorylated MAPK members p38 and Erk1/2 in HCC1937 cells (Fig. 5C). The pharmaceutical inhibition of GRK6 kinase activity by GRK6-IN-2 was found to dose-dependently reduced the protein levels of phosphorylated p38 and Erk1/2 in MDA-MB231 cells (Fig. 5D). Accordingly, the block of β-Arrestin activity by Barbadin dose-dependently suppressed the GRK6-elevated protein phosphorylation of p38 and Erk1/2 in HCC1937 cells (Fig. 5E). The inhibition of p38 and Erk1/2 kinase activity by SB203580 and U0126, respectively, abrogated the GRK6-forced EMT progression (Fig. 5F) and cellular migration ability (Fig. 5G) in HCC1937 cells.

**Fig. 6:**
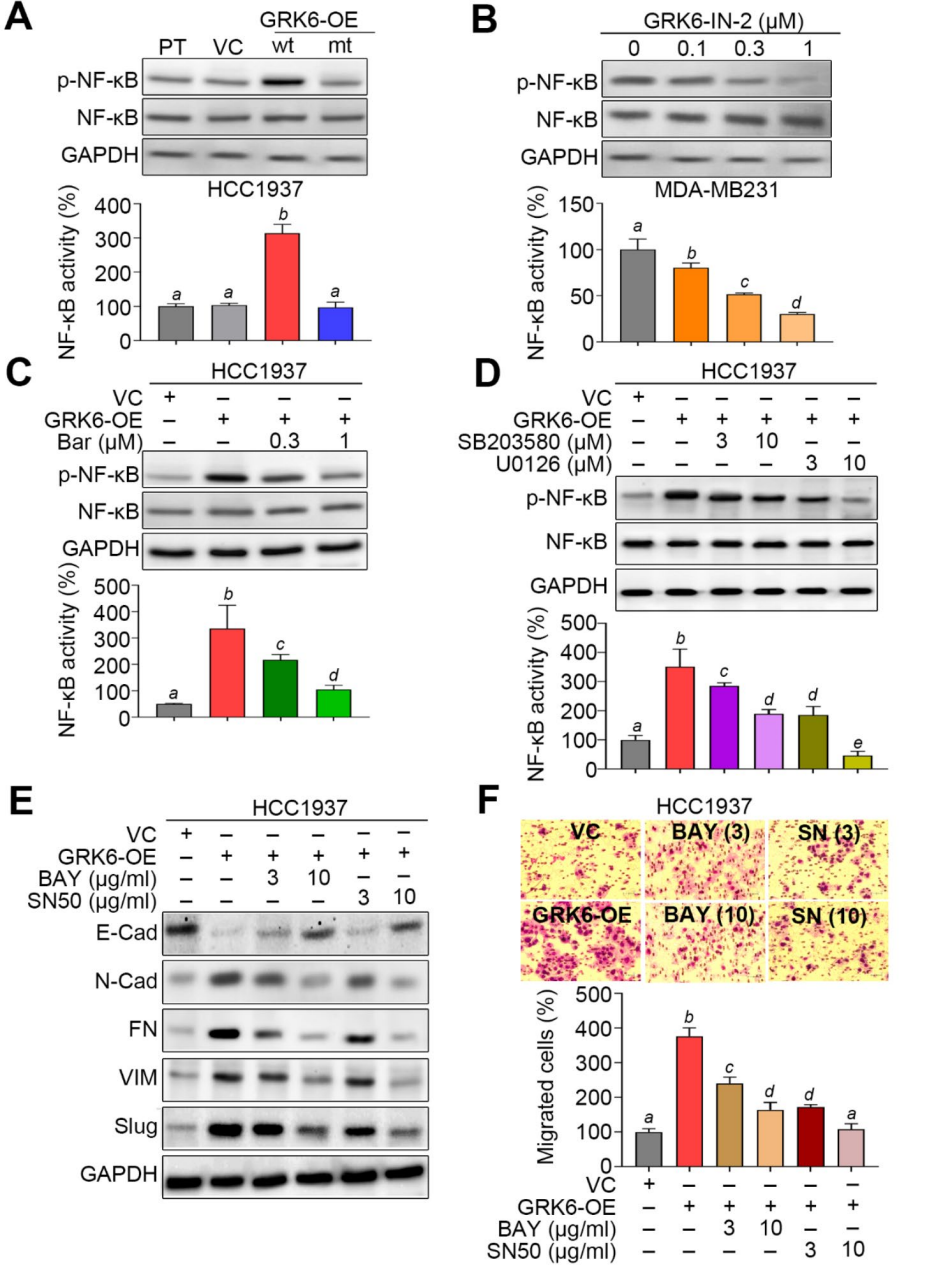
The GRK6/β-Arrestin/MAPK signalling axis induces NF-κB transcription factor activity to force the EMT progression and cellular migration activity in TNBC cells. **A–D.** Western blot analyses (upper) for the p-NF-κB, total NF-κB and GAPDH in the whole cell lysates and luciferase reporter assays (lower) for the NF-κB DNA-binding activity in the indicated HCC1937 cell variants (A), MDA-MB231 cells treated without or with GRK6 inhibitor at the indicated concentrations (B), and the VC/GRK6A-OE HCC1937 cells treated without or with β-Arrestin inhibitor (C) and MAPK inhibitors (D) at the indicated concentrations for 24 h. **E–F.** Western blot analyses for the EMT markers in the whole cell lysates (E), and Giemsa stain for the migrated cells in the trans-well cultivation (F, upper) of VC and GRK6A-OE HCC1937 cells treated without or with NF-κB inhibitors BAY-11-7082 (BAY) and SN50 at the indicated concentrations for 24 h. The migration cell number from three independent experiments were presented as mean ± SEM in the histogram (F, lower). The different alphabets indicate the statistical significance at *p* < 0.05. In A, B, C, D and E, GAPDH was used as an internal control of protein loading

Transcriptional activity of NF-κB is tightly regulated by MAPK and required for the expression of EMT-related genes [23]. On the other hand, we performed another GSEA experiment using the ranked somatic genes by Spearman’s rho values derived from GRK6 co-expression in TCGA TNBC samples (Fig. S5A) against Hallmark gene sets. The data showed that GRK6 expression positively and significantly (*p* < 0.05) correlates with the gene sets of TNFA_SIGNALING_VIA_NFKB and EPITHELIAL_MESENCHYMAL TRANSITION (Fig. S5B). Based on these findings, we further determined its roles in the GRK6-promoted metastatic progression in TNBC. We found that wild-type, not mutant, GRK6 overexpression robustly induces the protein phosphorylation and DNA-binding activity of NF-κB in HCC1937 cells (Fig. 6A). Similarly, the treatment with GRK6 kinase inhibitor GRK6-IN-2 dose-dependently suppressed the protein phosphorylation and DNA-binding activity of NF-κB in MDA-MB231 cells (Fig. 6B). Moreover, the inclusion of β-Arrestin, p38 and Erk1/2 inhibitors Barbadin, SB203580 and U0126 respectively reduced the GRK6-enhanced protein phosphorylation and DNA-binding activity of NF-κB in HCC1937 cells in a dose-dependent manner (Fig. 6C and D). Finally, the treatment with NF-κB inhibitors BAY-11-7082 and SN50 markedly suppressed the GRK6-enhanced EMT progression (Fig. 6E) and cellular migration ability (Fig. 6F) in HCC1937 cells. Accordingly, SN50 treatment dramatically suppressed lung colony-forming ability of GRK6-overexpressing HCC1937 cells in a dose-dependent manner (Figure S8A and B).

### Pharmaceutical inhibition of GRK6 suppresses lung metastasis of TNBC

**Fig. 7:**
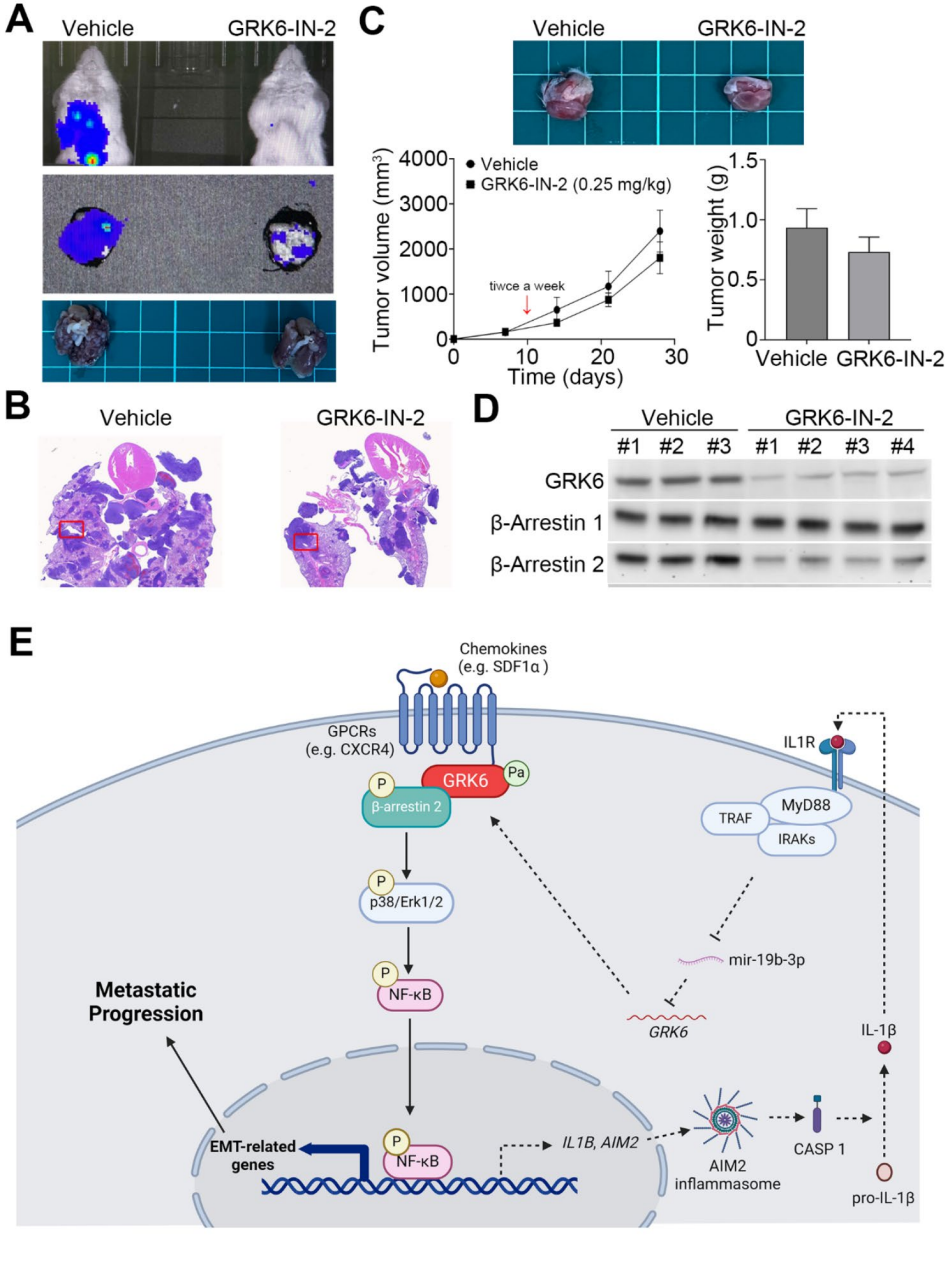
Targeting GRK6 potentially suppresses lung metastasis of TNBCs. **A** Luminescent intensity (upper and middle) and tumor nodules (lower) of luciferase-expression MDA-MB-231 cells in the lungs of tumor-bearing mice treated without (Vehicle, *n* = 3) or with GRK6-IN-2(*n* = 4) at 0.25 mg/kg twice a week. **B** H & E staining for the colonies of the MDA-MB-231 cell variants without with GRK6-IN-2 administration. **C** Tumor appearance, volume and weight of the MDA-MB-231 cell variants without with GRK6-IN-2 administration. **D** Western blot analysis for the protein expression of GRK6, β-arrestin 1, β-arrestin 2, p-Erk1/2, Erk1/2, p-NF-κB, NF-κB and GAPDH. GAPDH was used as an internal control of protein loading. **E** A proposed mechanism for the GRK6-promoted metastatic progression in TNBC

To estimate if targeting of GRK6 activity is capable of suppressing lung metastasis of TNBC, we performed orthotopic mouse model by injecting a lung metastatic MDA-MB231 cell variant which were established in our previous report [24] into mammary fat pad. Our data showed that the administration of GRK6 inhibitor GRK6-IN-2 at 0.25 mg/kg twice a week dramatically suppressed the lung metastasis (Fig. 7A and B) and slightly inhibited tumor growth (Fig. 7C) of the MDA-MB231 cell variant. Moreover, the treatment with GRK6-IN-2 predominantly reduced the protein expression of GRK6 and β-arrestin 2, not β-arrestin 1, and the phosphorylated levels of Erk1/2 and NF-κB in the primary tumors of MDA-MB231 cell variant (Fig. 7D).

## Discussion

Here we show that GRK6 upregulation is extensively detected in the primary tumors compared to normal mammary tissues and refers to a higher risk for distant metastasis in TNBC patients. Intriguingly, primary tumors from breast cancer patients with lymph node metastasis exhibited an increased membrane-bound GRK6. GRK6 knockdown dramatically reduced but overexpression predominantly enhanced the metastatic potentials of TNBC cells in vitro and in vivo. Our data further showed that the palmitoylated GRK6 which probably interacts with an agonist (e.g. SDF1α)-activated G protein-coupled receptors (GPCRs), e.g. CXCR4 (Ref), recruits its downstream effector β-Arrestin 2 to transduce the signal to the p38/Erk1/2/NF-κB pathway. Subsequently, the activated NF-κB triggers the transcription of EMT-related genes to force the metastatic progression of TNBC (Fig. 7E). On the other hand, the recent report demonstrated that the secreted IL-1β by proteolytic cleavage by Caspase 1 in the inflammasome (e.g. AIM2) complex restrains the post-translational inhibition of mir-19b-3p on GRK6 via eliciting the signal transduction of its receptor [25], thereby reinforcing the GRK6-mediated cellular responses. These findings provide a novel mechanism by which GRK6 promotes the metastatic potentials of TNBC via coordinating with inflammasome/IL-1β signaling pathway.

Palmitoylation of GRK6 at the C-terminal cysteine residues is key step for its membrane localization and protein kinase activity [20]. Indeed, our results indicated that the enforced expression of palmitoylatable, not non-palmitoylatable, GRK6 variant is capable of recruiting its downstream effector β-Arrestin 2 to cell membrane, activating the p38/Erk1/2-NF-κB signaling axis and triggering EMT and metastatic progression in TNBC cells. Two GRK6 spliced variants that lacks C-terminal palmitoylation sites have been identified [26]; however, their oncogenic roles remain unknown. Recently, GRK6 inhibitors, GRK6-IN-1 (compound 18) and GRK6-IN-2 (compound 10a), were chemically synthesized and suppressed the cell growth of multiple myeloma cells with a 50% of inhibitory concentration (IC_50_) at sub-µM [27]. Although GRK6-IN-1 was shown to be more effective and selective on inhibiting GRK6 activity [27], our data revealed that the treatment with GRK6-IN-2 at sub-µM predominantly suppresses the intracellular β-Arrestin 2, p38, Erk1/2 and NF-κB activity and the cellular migration ability and lung metastasis of TNBC cells. Moreover, IHC experiment reveal that the increased membrane-bound GRK6 levels likely associates with lymph node metastasis even though this phenomenon is still needed to be validated in the larger cohorts. It implicates that targeting the palmitoylation of GRK6 could be a new therapeutic strategy to combat metastatic TNBC in the future clinics.

The activation of β-Arrestin 1/2 has been associated with molecular mechanism for chemoresistance in breast cancer [28, 29]. As a result, β-Arrestin was thought to be a selective target in breast cancer treatment [30]. Moreover, the protease-activated receptor 2-mediated migration of MDA-MB231 cells was shown to require the activation of β-Arrestin 1/2 [31]. Nevertheless, the overexpression and knockdown of β-Arrestin 1 was found to inversely regulate the migration ability of MDA-MB231 cells [32]. In contrast, the SUMOylation deficiency of β-Arrestin-2 resulted in slower migration of MDA-MB231 cells [33]. These findings may implicate the critical role of β-Arrestin-2 in regulating the metastatic potentials of TNBC cells. Here we further show that the membrane localization and activation of β-Arrestin 2, not β-Arrestin 1, by the activated/membrane-associated GRK6 is required for triggering the metastatic progression of TNBC cells. Since β-Arrestin 2 recruitment and activation could be also induced by other GRKs, e.g. GRK2, in response to the chemokine receptor CXCR4/7-potentiated cell migration [22], the identification of GPCR that triggers the activation of GRK6/β-Arrestin 2 axis is critical for the management of TNBC metastasis. Besides, the opposite effects of GRK3 on the CXCR4-triggered metastatic progression of breast cancer [18] is needed to be further validated in the GRK6-promoted TNBC metastasis. Further experiments are also required for understanding the crosstalk between the GRK4/β-Arrestins [19] and GRK6/β-Arrestin 2 pathway-fostered TNBC metastatic progression through the activation of MAPK family.

The activation of MAPK by either β-Arrestin 1 or β-Arrestin 2 is commonly found in the GPCR-induced intracellular signalling pathway [34, 35]. Recent reports demonstrated that β-Arrestin 2 triggers the activation of Erk1/2 pathway to promote melanoma and colorectal cancer metastasis [36, 37]. Moreover, β-Arrestin 2 was shown to regulate glucose metabolism and inflammatory signalling via activating p38 MAPK pathway [38, 39]. The co-activation of Erk1/2 and p38 MAPK by β-Arrestin 2 was found to prevent cell apoptosis [40]. Here we further find that GRK6-activated β-Arrestin 2 induces the activation of both MAPK members to enhance the metastatic capacity of TNBC cells. Although β-Arrestin 2 frequently activates Erk1/2 and p38 MAPK pathways upon the activation of GPCRs, the activation of JNK, another MAPK member, by β-Arrestin 2 was also detected under the stimulation of angiotensin II type 1 A receptor [41]. As a consequence, the β-Arrestin 2-mediated JNK activation is needed to be further pursued in the GRK6-triggered metastatic progression of TNBC.

NF-κB has been shown to mediate EMT process in colorectal cancer [42], gastric cancer [43] and TNBC [44]. Moreover, the activation of NF-κB was also closely associated with the metastatic progression in several types of cancer [45–50]. In breast cancer, NF-κB played a pivotal role in triggering cancer initiation, growth, metastasis and resistance to chemotherapy [51]. Here we further show that NF-κB is sequentially activated by GRK6, β-Arrestin 2 and MAPKs and mediates the expression of EMT-related genes and metastatic progression in TNBC cells. Although further experiments are still needed to investigate if GRK6 plays a central role in regulating NF-κB activity upon the activation of GPCRs, e.g. chemokine receptor CXCR4 in TNBC [52], our results provide a novel mechanism in which GRK6 sequentially activates β-Arrestin 2 and MAPKs to force the transcriptional activity of NF-κB and thereby triggers the EMT progression and metastasis in TNBC.

## Conclusions

Our results indicate that GRK6 upregulation is extensively found in primary tumors compared to normal mammary tissues and refers to a poor prognosis probably due to a higher risk for distant metastasis in TNBC. Moreover, our data demonstrated that the palmitoylated and membrane-associated GRK6 recruits β-Arrestin 2 to activate MAPK/NF-κB signalling axis and ultimately force the metastatic progression of TNBC. These findings may provide a new therapeutic strategy to combat metastatic TNBCs by targeting GRK6 activity.

## Electronic supplementary material

Below is the link to the electronic supplementary material.

Supplementary Material 1

## Data Availability

The data and materials generated in this study are available upon reasonable request from the corresponding author.

## Abbreviations

TNBC: Triple negative breast cancer
GRK6: G protein–coupled receptor kinase 6
MAPKs: Mitogen-activated protein kinases
ER: Estrogen receptor
PR: Progesterone receptor
HER2: Human epidermal growth factor receptor 2
GPCR: G protein-coupled receptor
