# Supplementary material for: GRK6 palmitoylation dictates triple-negative breast cancer metastasis via recruiting the β-Arrestin 2/MAPKs/NF-κB signaling axis
Source: Breast Cancer Res. 2024 Dec 31;26:193. doi: 10.1186/s13058-024-01953-z (PMC11689595; doi:10.1186/s13058-024-01953-z)
Supplement: Supplementary file 1 — Supplementary Material 1 [file 13058_2024_1953_MOESM1_ESM.docx]

**Supplementary Information**

**
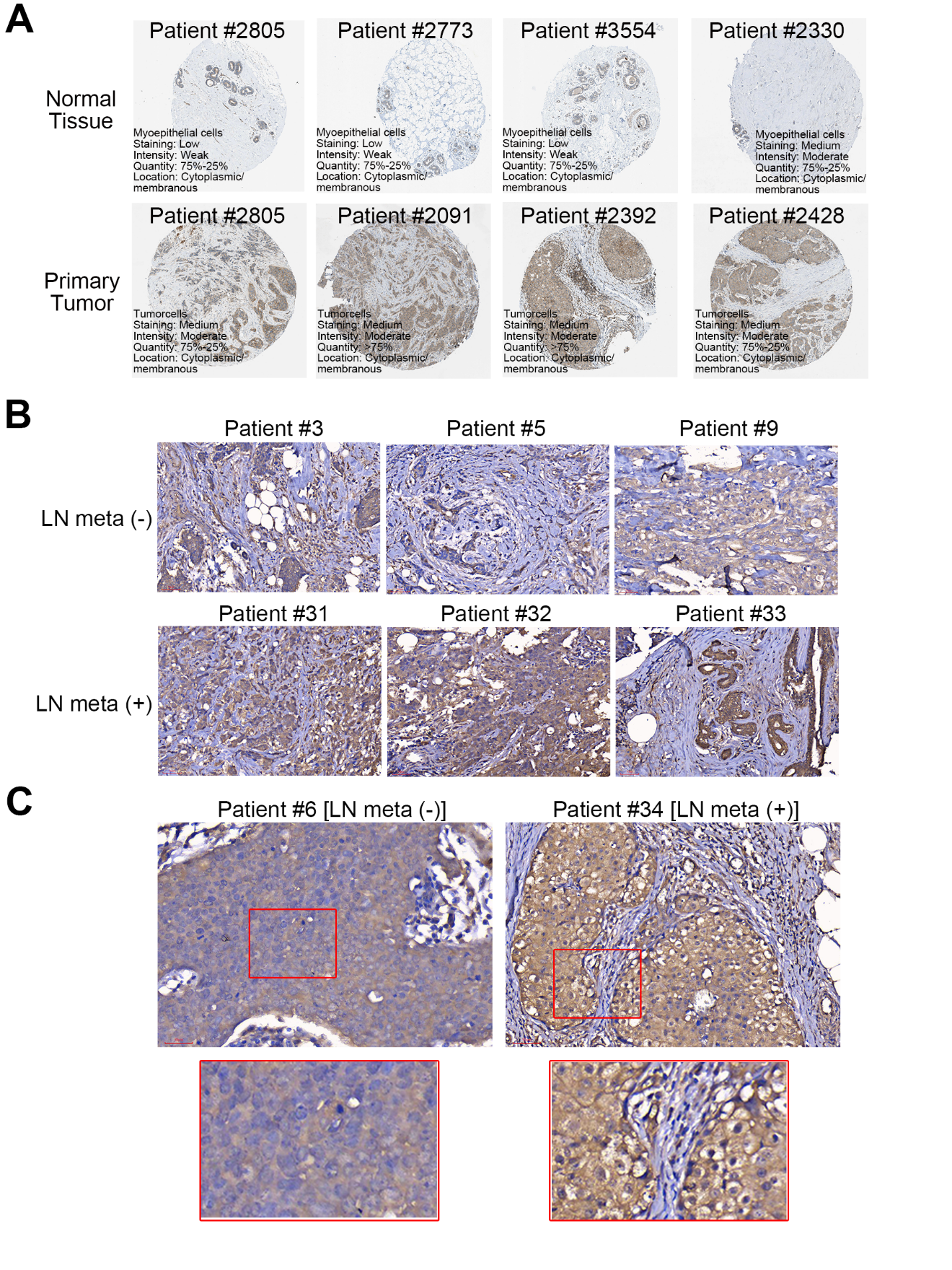
**

**Figure S1.** (A - C) The results of IHC staining for GKR6 protein against normal tissues and primary tumors of breast cancer patients from The Human Protein Atlas database (A) and the primary tumors derived from breast cancer patients without or with lymph node metastasis (LN meta, B and C).


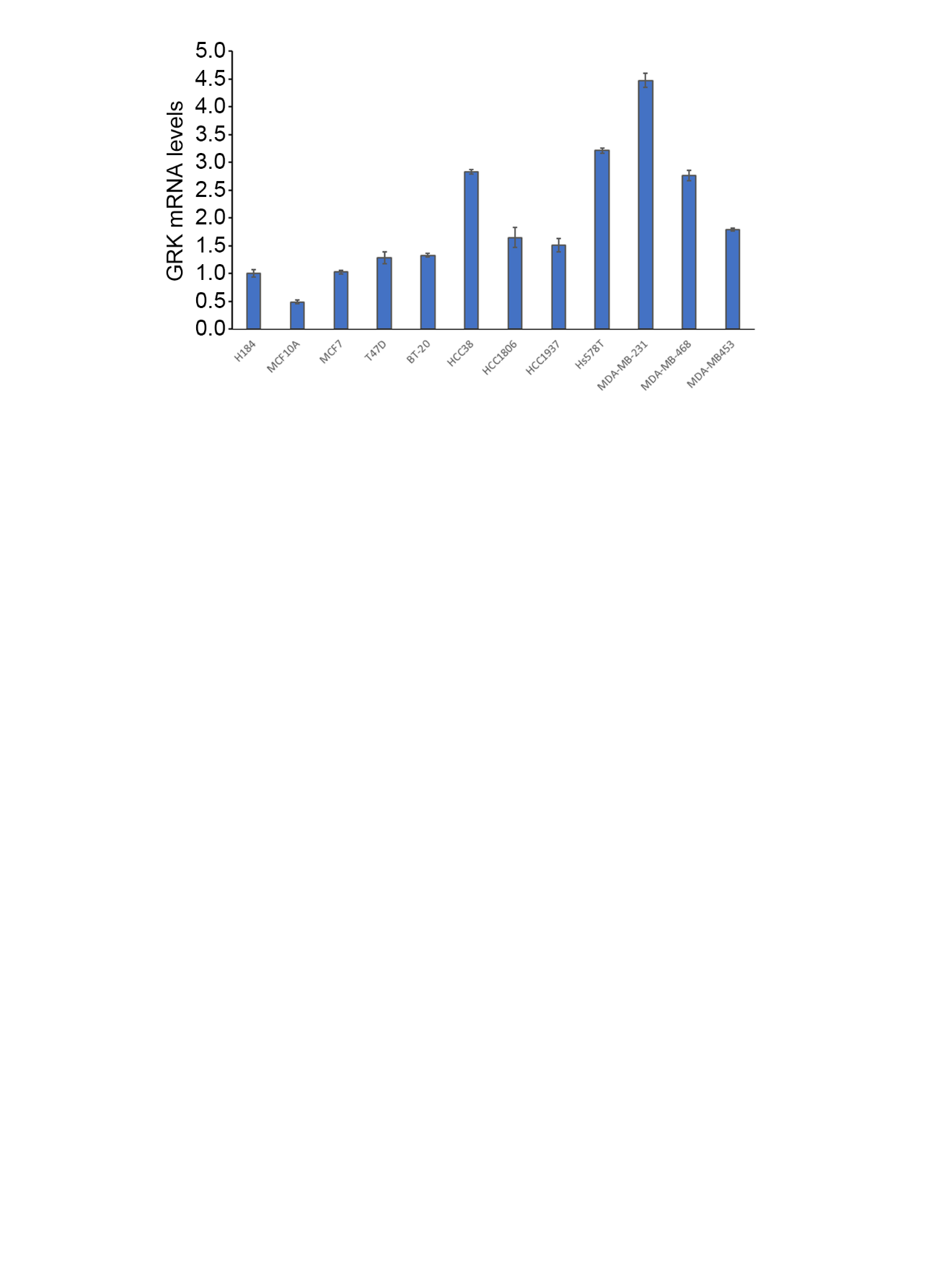


**Figure S2.** The quantitative RT-PCR for detecting the mRNA levels of GRK6 in the non-turmor mammary cell lines H1845F5 (H184) and MCF10A, ER+ cell lines MCF7, T47D and BT-20, and TNBC cell lines HCC38, HCC1806, HCC1937, Hs578T., MDA-MB231, MDA-MB468 and MDA-MB453.

**
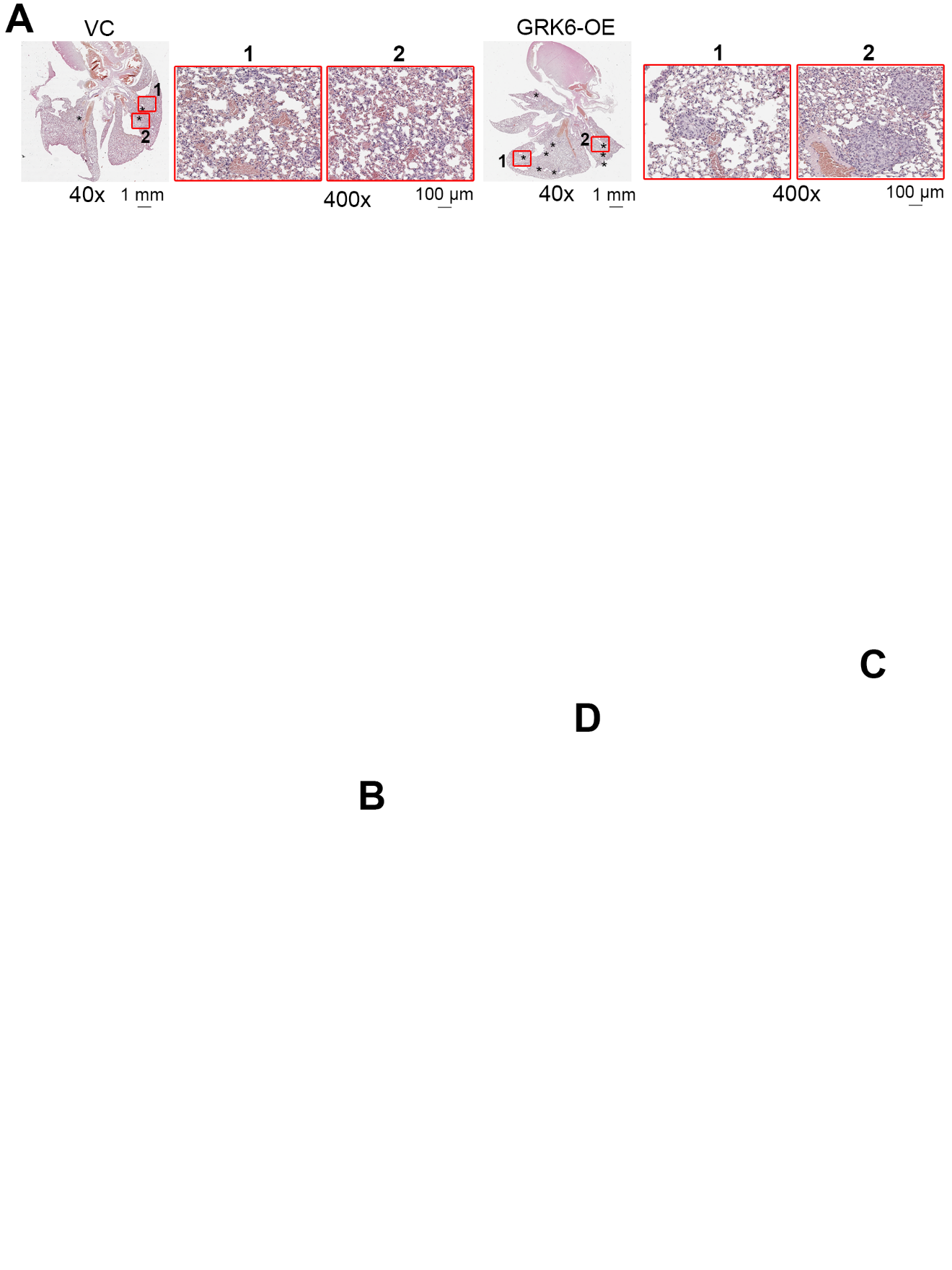
**

**Figure S3. The additional view of H&E staining of Figure 2H**

**
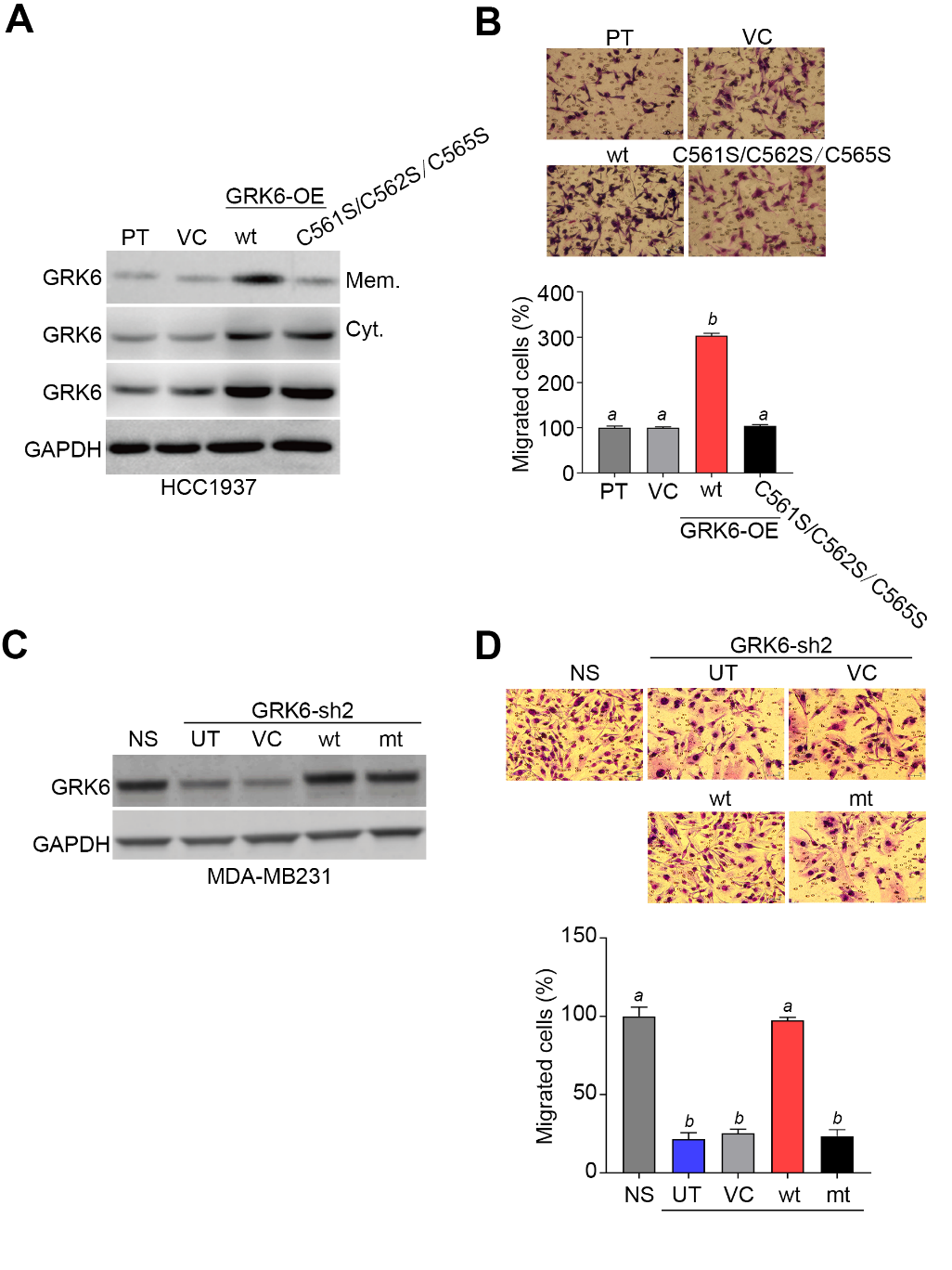
**

**Figure S4.** (A) Western blot analysis for determining the protein levels of GRK6 and GAPDH in the parental, vector control (VC) and the wild-type (wt) and C561S/C562S/C565S-mutant GRK6-overexpressing MDA-MB231 cells (B) Giemsa stain for the migrated cells in the trans-well cultivation (upper) of the indicated HCC1937 cell variants. The migration cell number from three independent experiments were presented as mean ± SEM in the histogram lower). The different alphabets indicate the statistical significance at p < 0.05. (C) Western blot analysis for determining the protein levels of GRK6 and GAPDH in the non-silencing (NS) control and GRK6-silenced MDA-MB231 cells without (vector control, VC) or with the restoration of wild-type (wt) or mutant (mt) GRK gene. (D) Giemsa stain for the migrated cells in the trans-well cultivation (upper) of the indicated MDA-MB231 cell variants. In B and D, the migration cell number from three independent experiments were presented as mean ± SEM in the histogram lower).

**
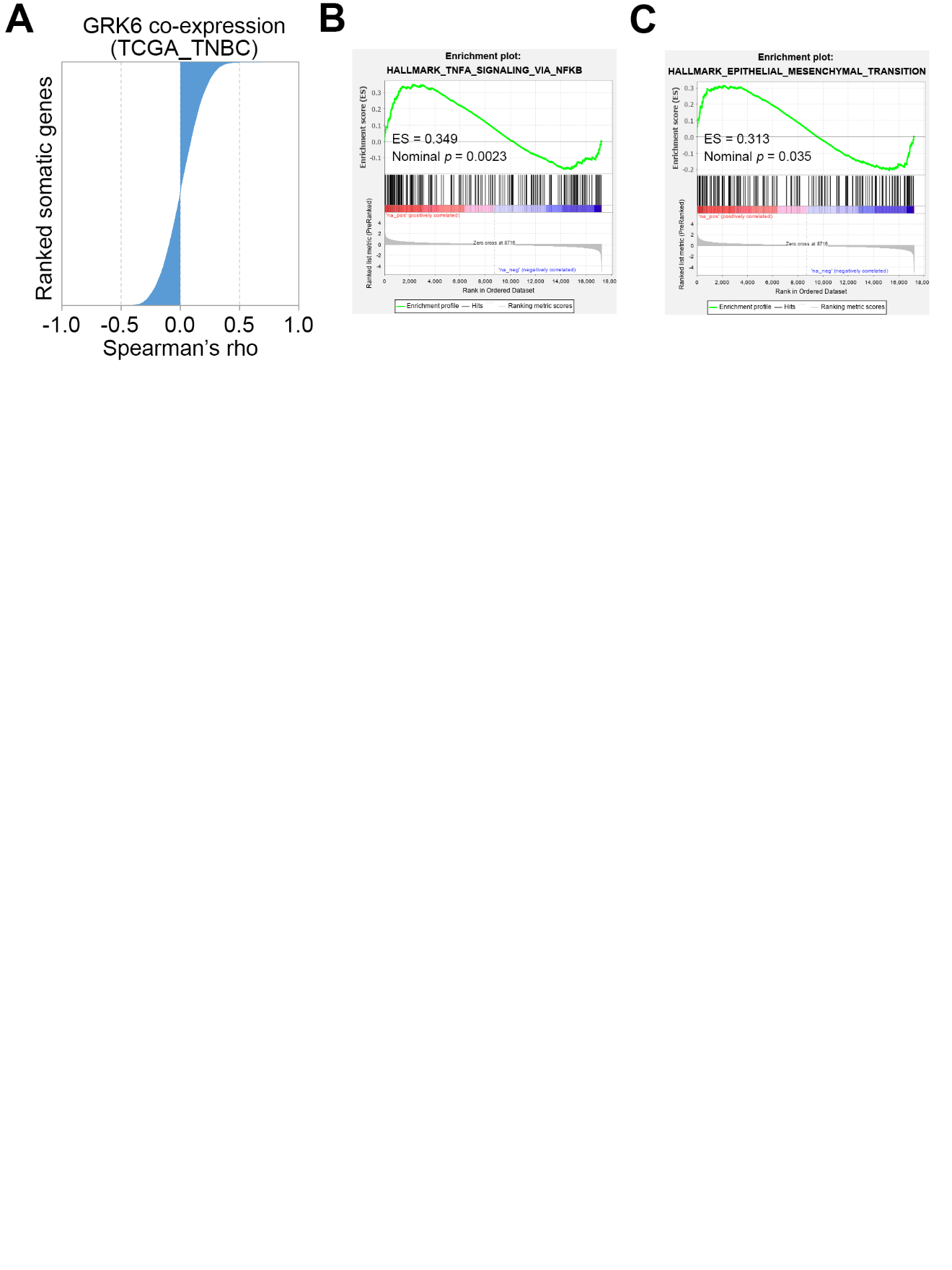
**

**Figure S5. GRK6 expression highly correlates with NF-κB activation and EMT progression in TNBC. A.** Bar chart presents the ranked somatic genes by Spearman’s rho values derived from the GRK6 co-expression in TCGA TNBC samples (n = 123). **B–C.** The plot of enrichment scores derived from GESA experiment against the ranked somatic genes shown in A and the gene sets of TNFA_SIGNALING_VIA_NFKB (B) and EPITHELIAL_MESENCHYMAL TRANSITION (C).

**
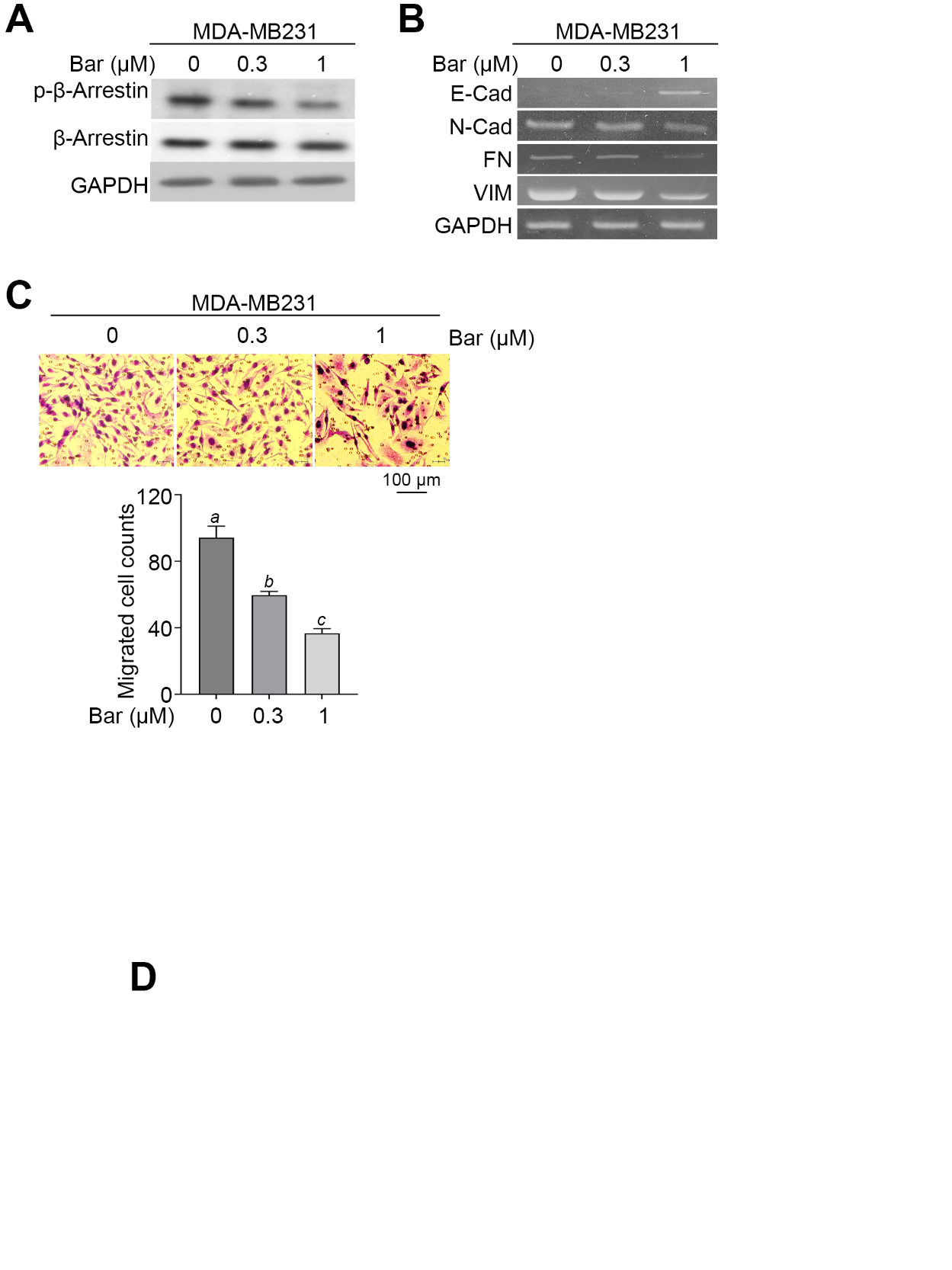
**

**Figure S6. The treatment with Barbadin suppresses β-Arrestin phosphorylation, EMT progression and cellular migration in the highly metastatic MDA-MB231 cells.** (A –C) Western blot analysis for determining the protein levels of phosphorylated β-Arrestin, total β-Arrestin and GAPDH (A), RT-PCR experiment for detecting the mRNA levels of EMT markers E-cadherin (E-Cad), N-Cad, Fibronectin (FN), Vimentin (VIM) and GADPH (B), and trans-well cultivation for measuring cellular migration ability (C) in the MDA-MB231 cells treated with Barbadin in the indicated concentrations. GAPDH was used as the internal control of experiments. The migrated cell number from three independent experiments were presented as mean ± SEM in the histogram (C, bottom). In C, the different alphabets indicate the statistical significance at p < 0.05 analysed by Kruskal–Wallis one-way analysis of variance.

**
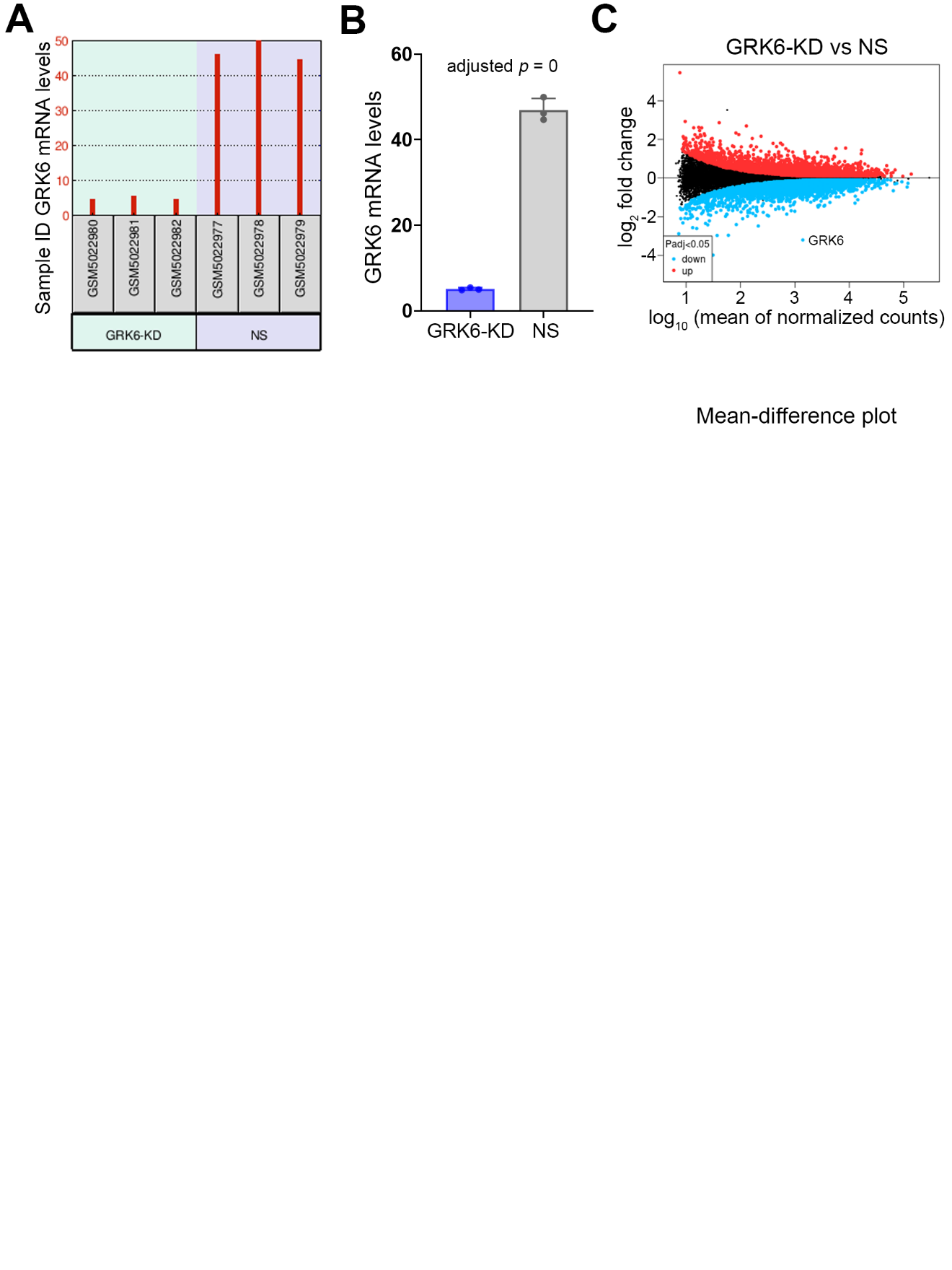
**

**Figure S7. GRK6 knockdown robustly represses its endogenous mRNA levels in ATII cells. A**. Histogram for the GRK6 mRNA levels detected by RNA-sequencing against three independent samples of GRK6-knockdown (GRK6-KD; GSM5022980, GSM5022981 and GSM5022982) and non-silencing (NS; GSM5022977, GSM5022978 and GSM5022979) control ATII cells. **B.** Histogram presents GRK6 mRNA levels as mean ± SEM from three independent samples of GRK6-KD and NS control ATII cells. Adjusted p value was obtained from GEO2R analysis. C. The mean-difference plot for log_10_ (mean of normalized counts) derived from mRNA levels of all genes determined by RNA-sequencing and log_2_ fold change from the difference of mRNA levels of all genes between the GRK6-KD and NS control ATII cells. The red and blue dots represent genes with a significant (adjusted *p* < 0.05) upregulation and downregulation, respectively.

**
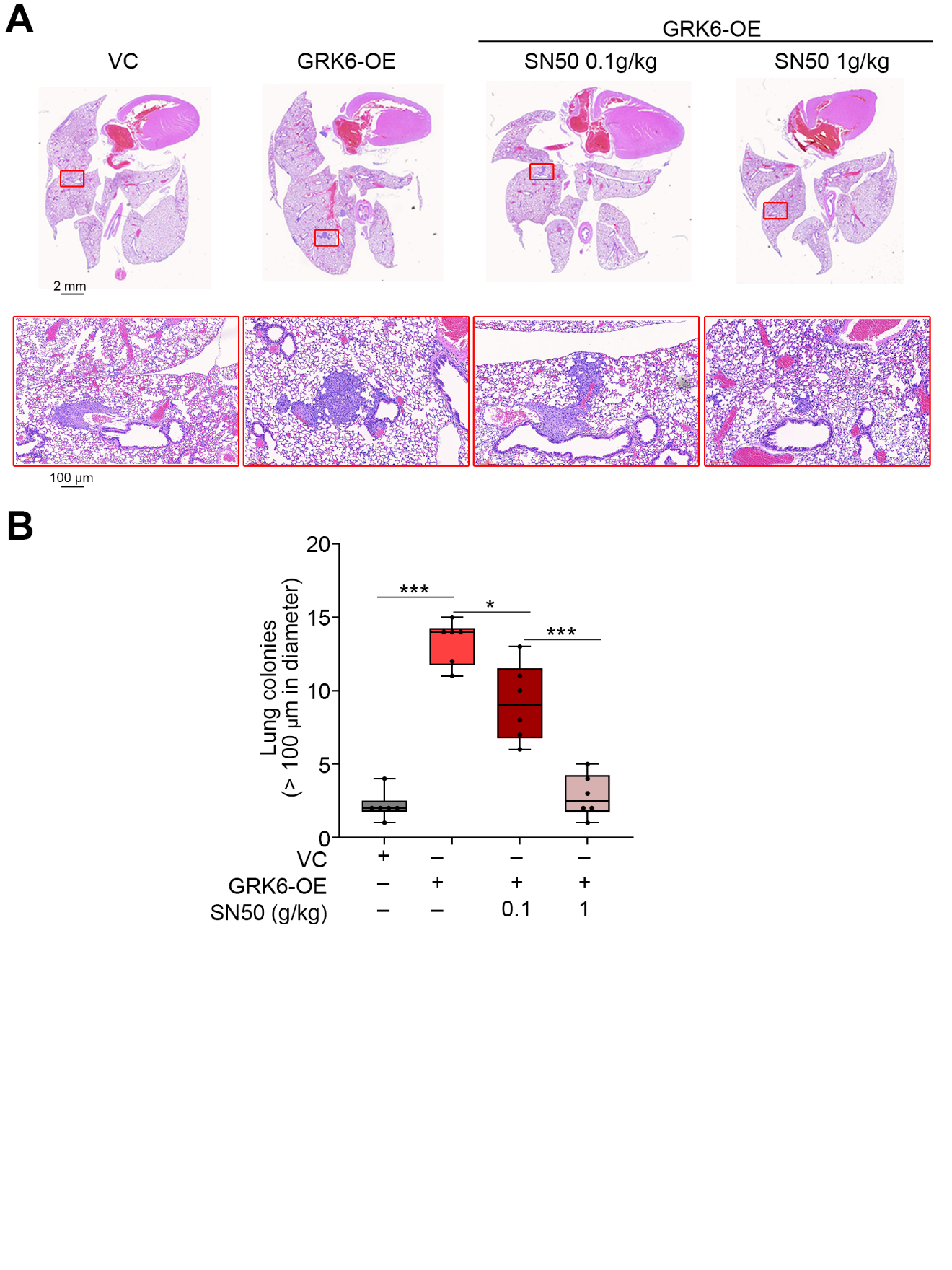
**

**Figure S8**. (A and B) H&E staining (A) and box plot (B) for lung colonies of the vector control from tumor-bearing mice (n = 6) and the GRK6-OE HCC1937 cell variants from tumor-bearing mice treated without or with SN50 at the indicated dosages twice a week after implantation for 2 weeks (n = 6 of each). The symbol “*” and “***” denote the statistical significance at p< 0.05 and p < 0.001, respectively.

**
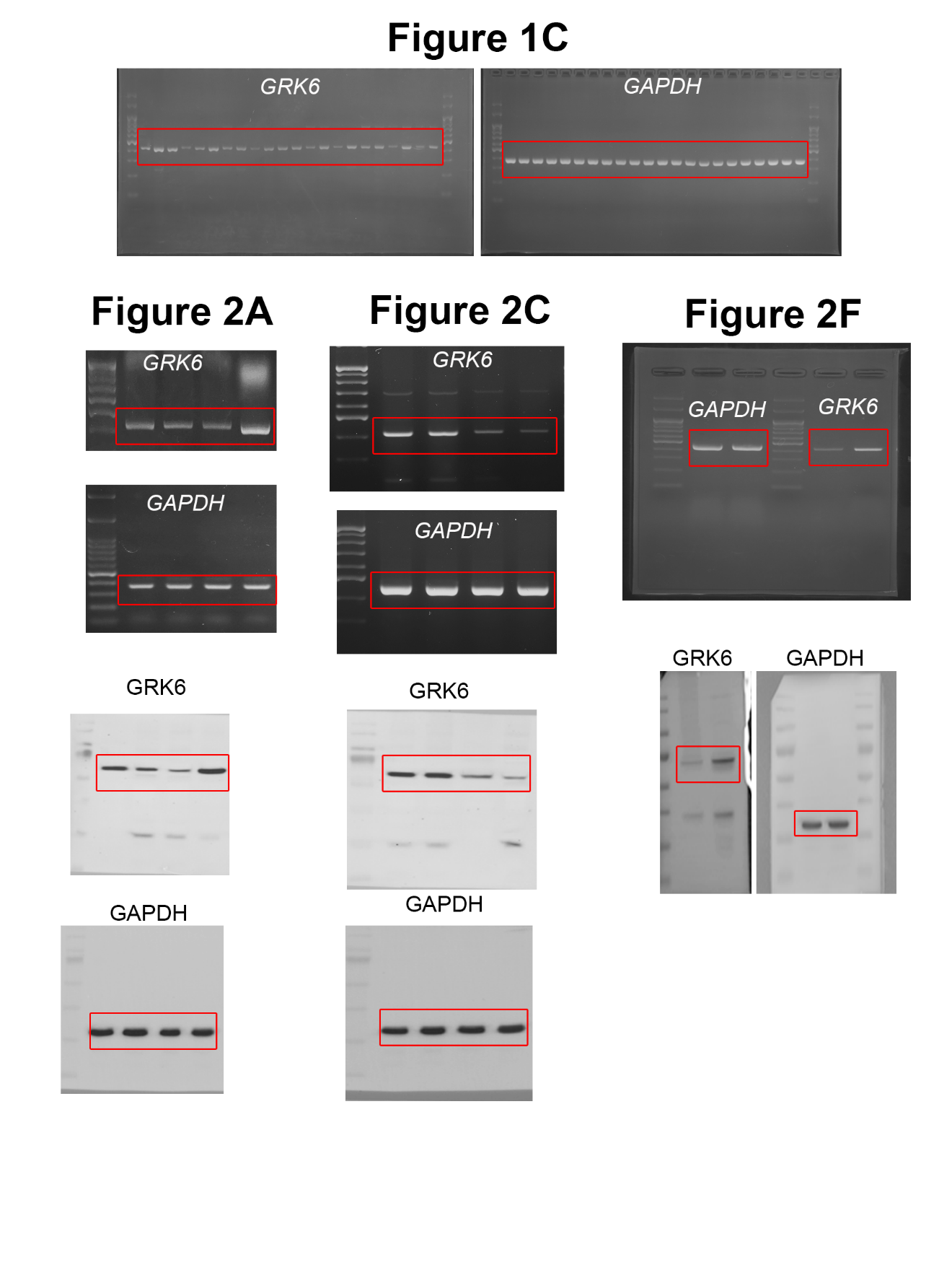
**

**Figure S9. The full uncropped Gels and Blots for Figure 1 and Figure 2.**

**
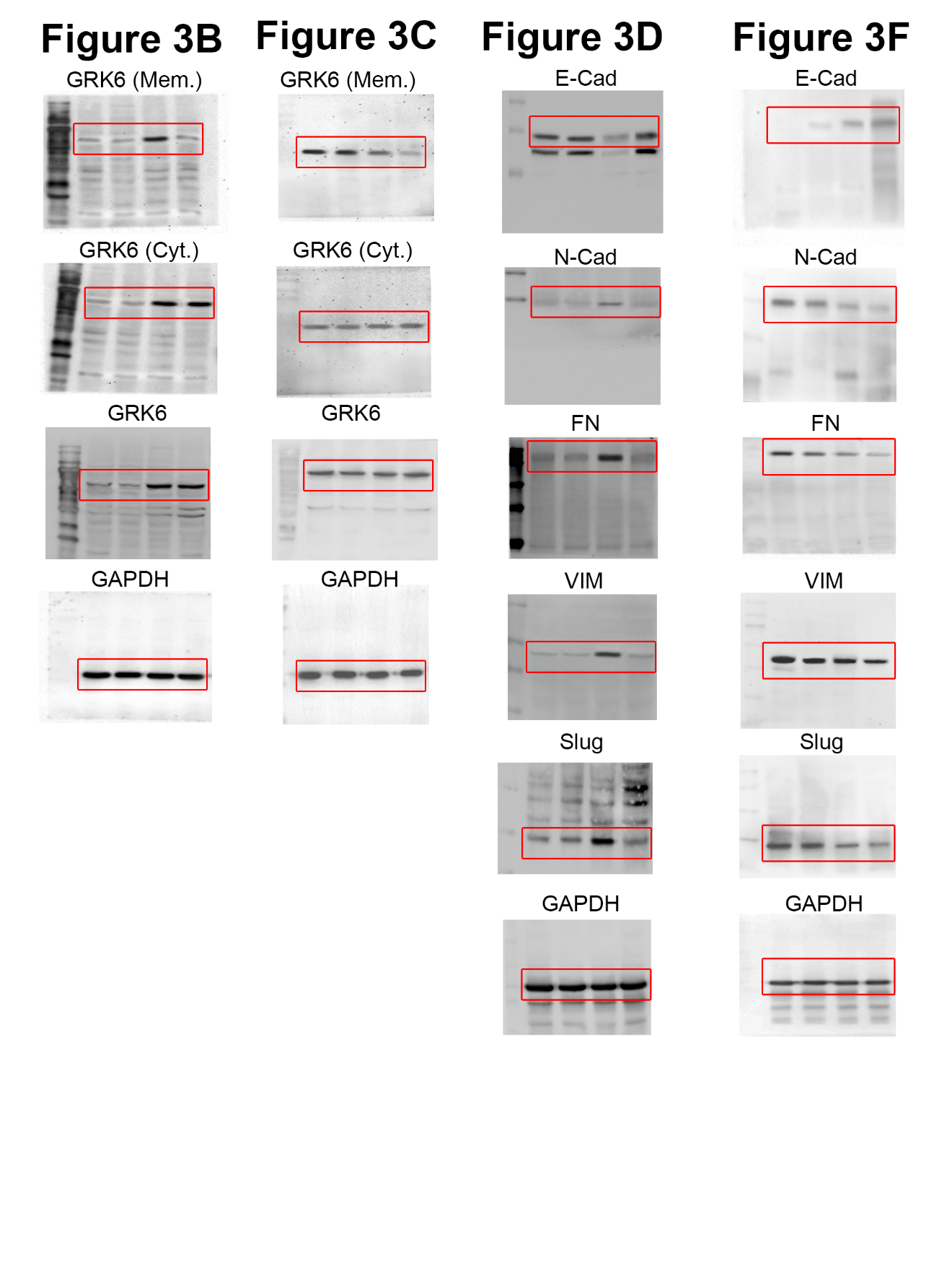
**

**Figure S10. The full uncropped Blots for Figure 3.**

**
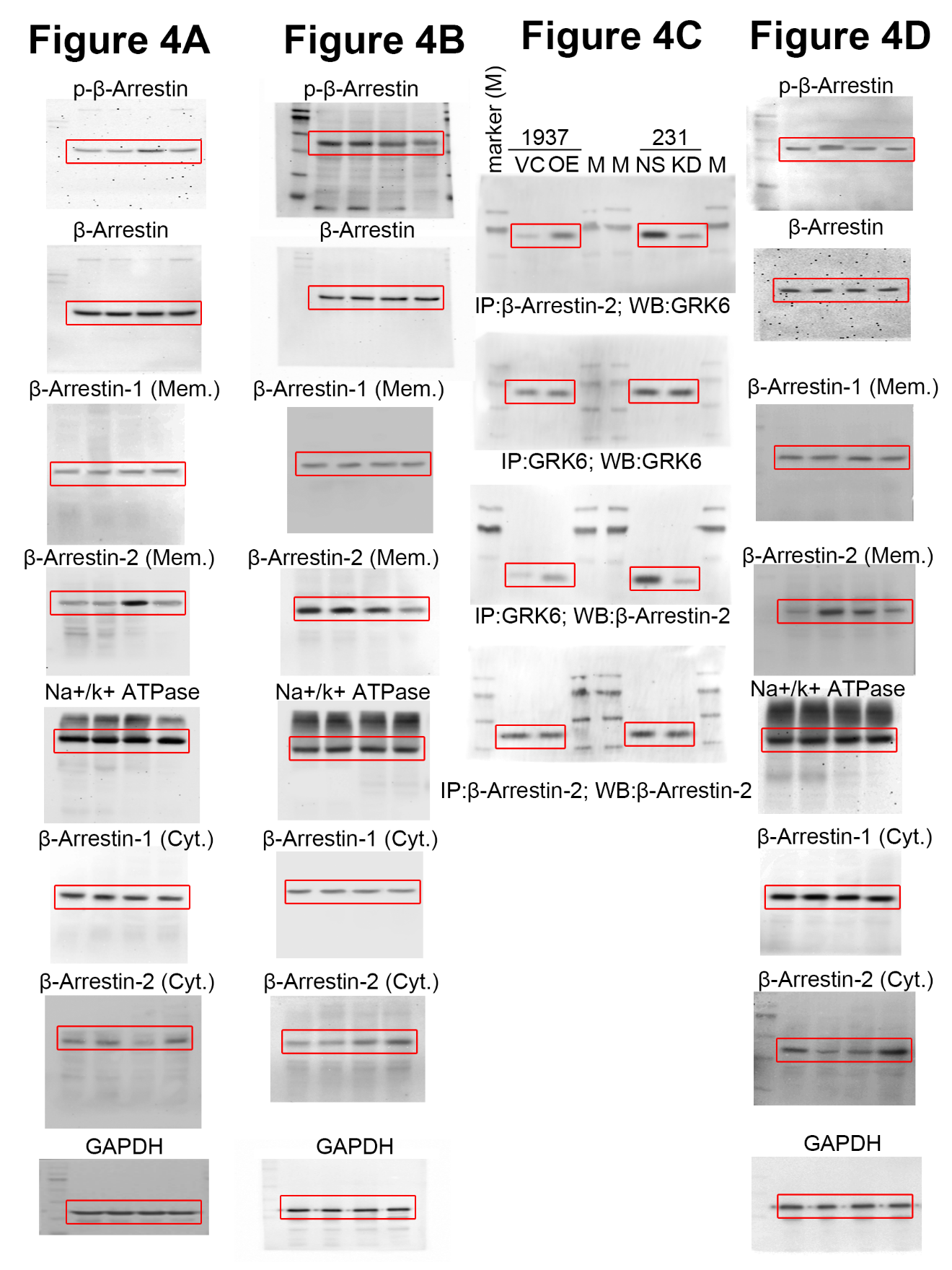
**

**Figure S11. The full uncropped Blots for Figure 4A – 4D.**

**
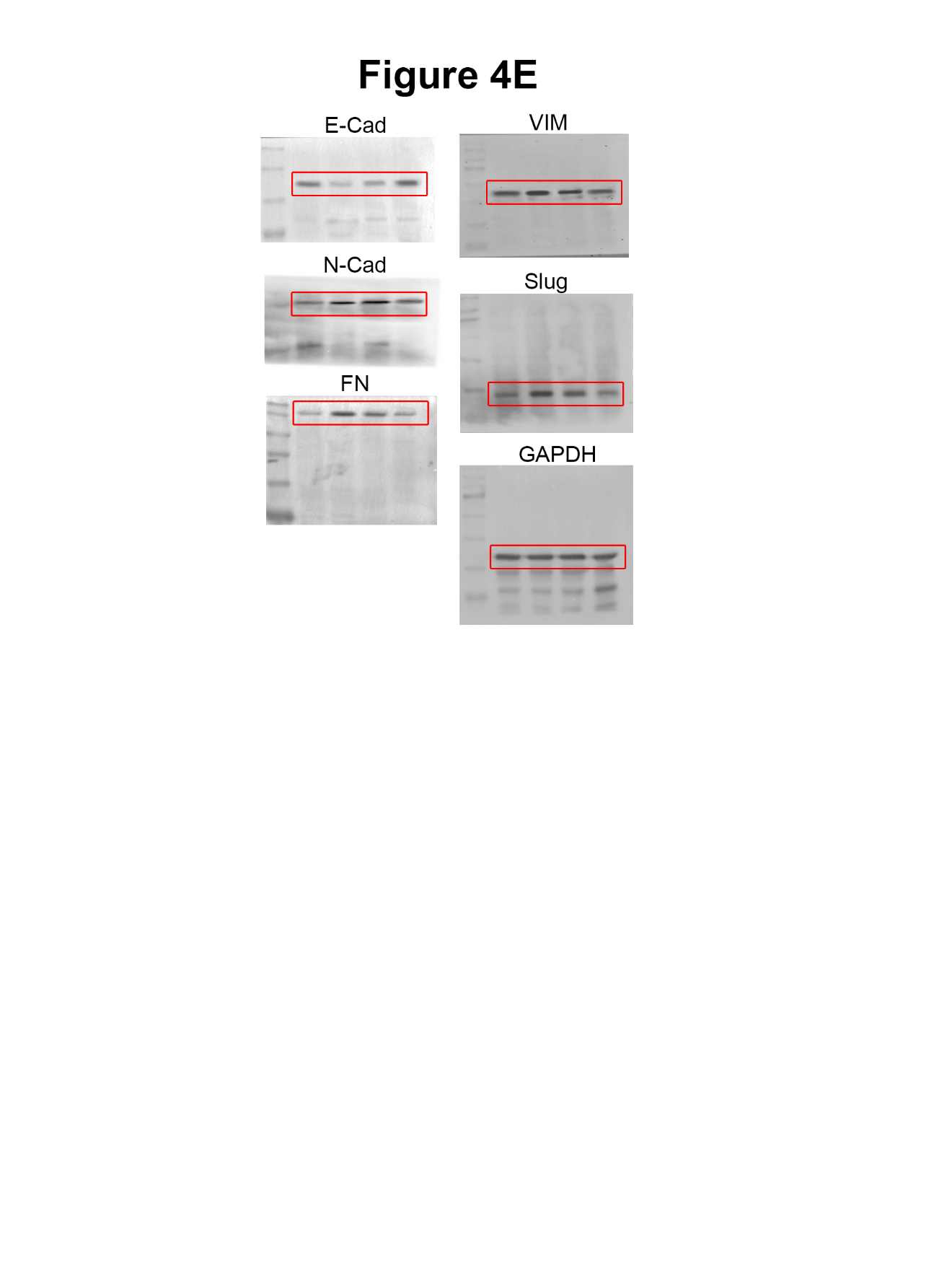
**

**Figure S11. The full uncropped Blots for Figure 4E.**

**
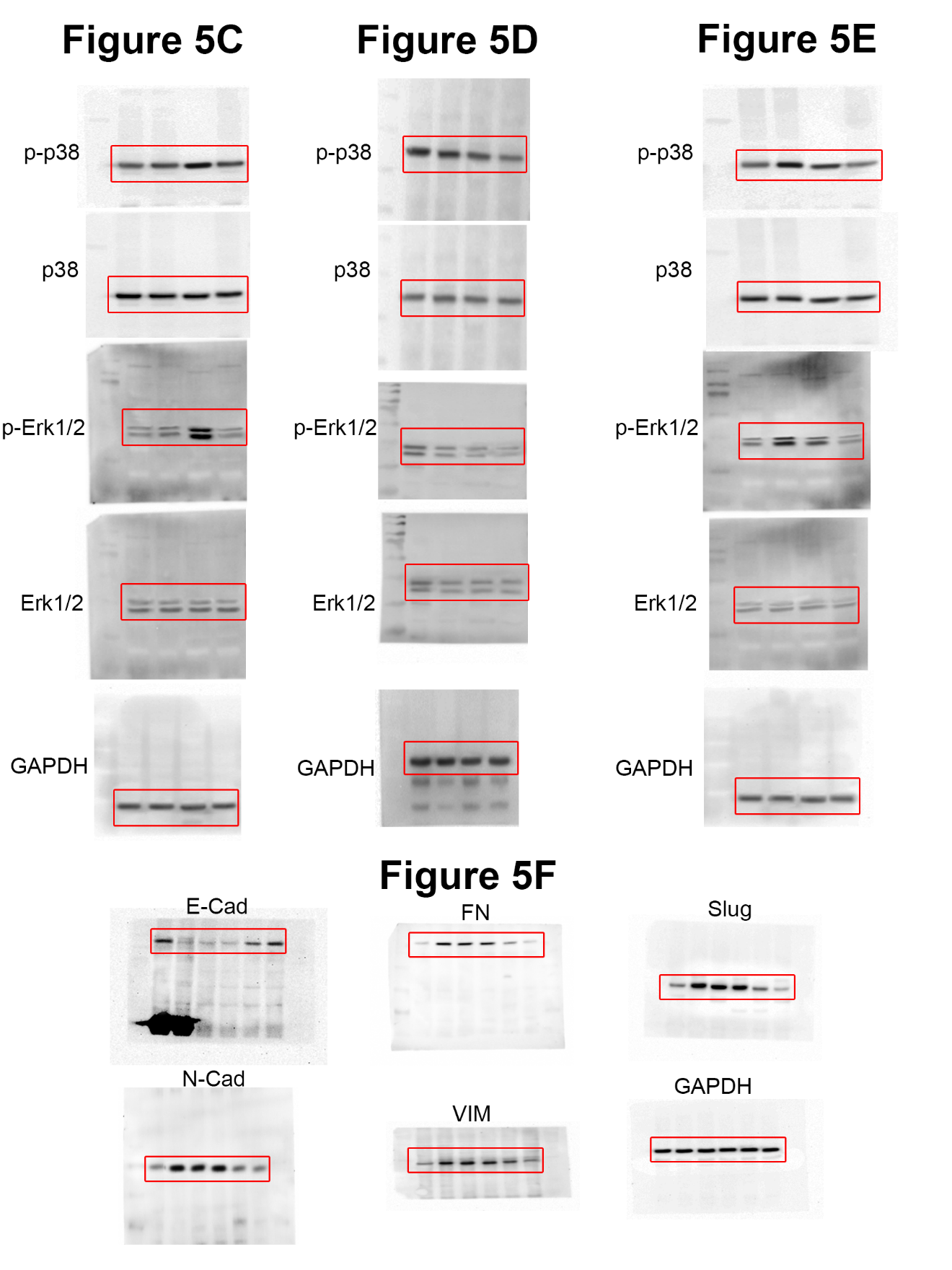
**

**Figure S13. The full uncropped Blots for Figure 5.**

**
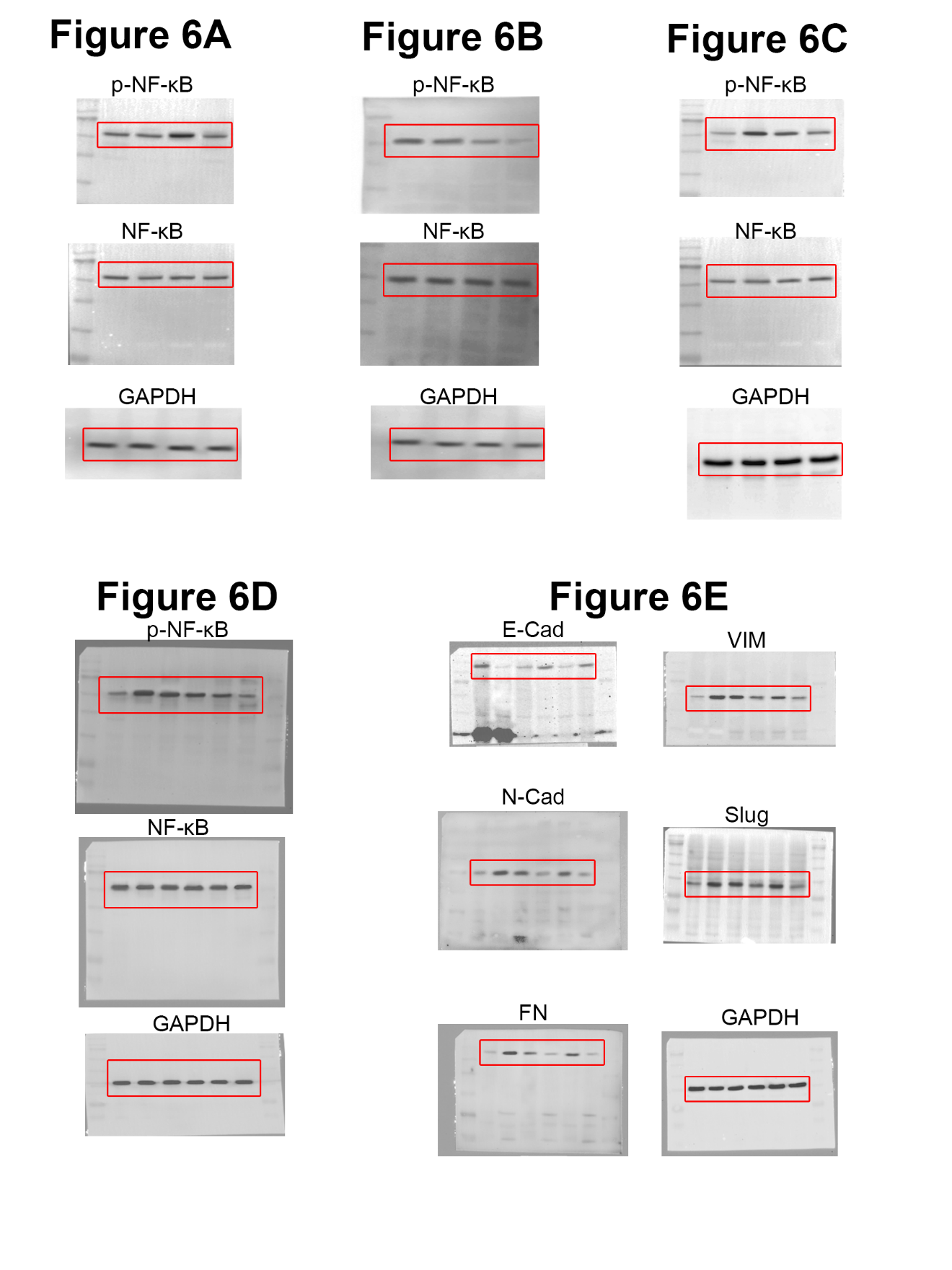
**

**Figure S14. The full uncropped Blots for Figure 6.**

**
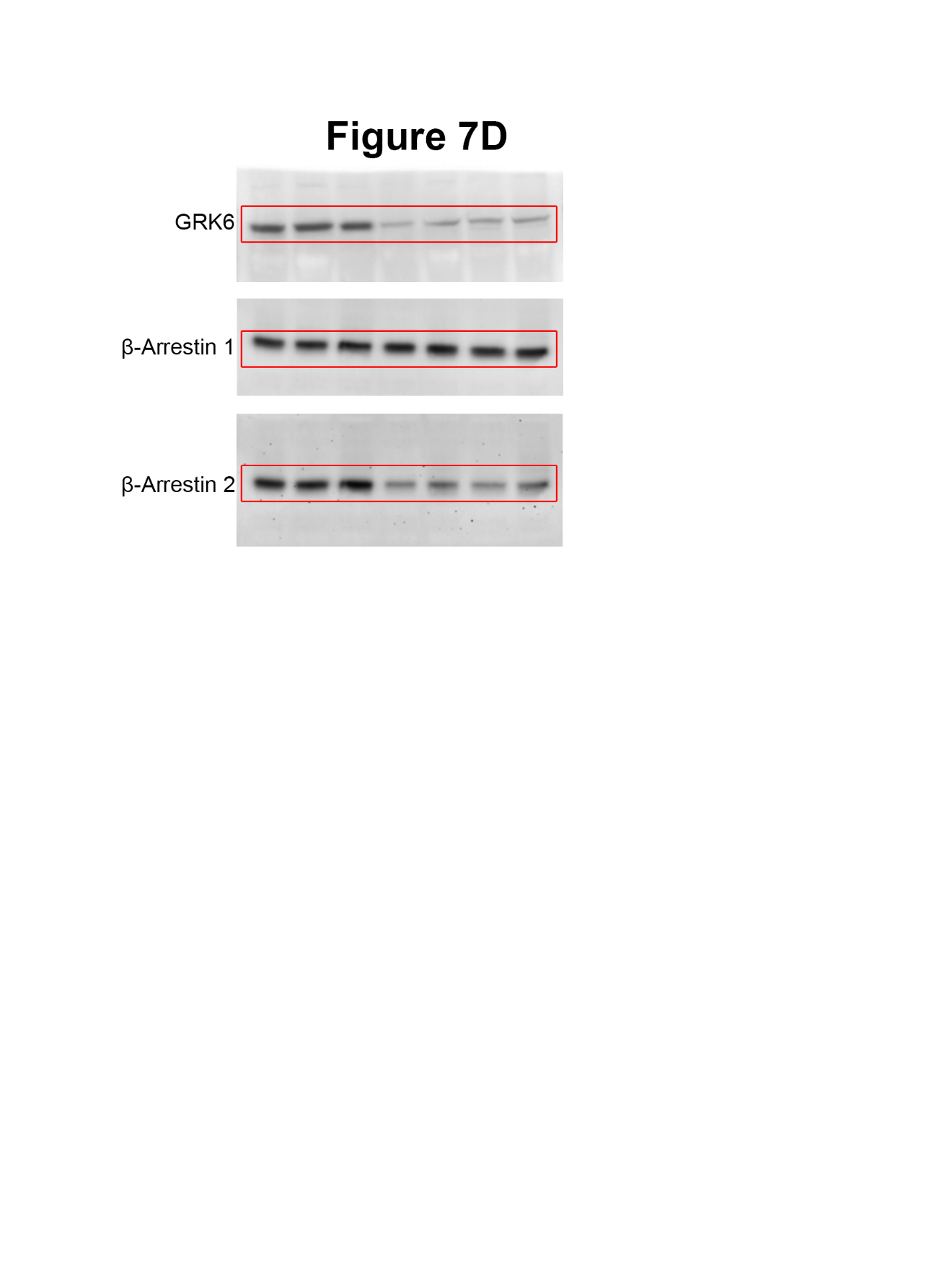
**

**Figure S15. The full uncropped Blots for Figure 7D.**

**
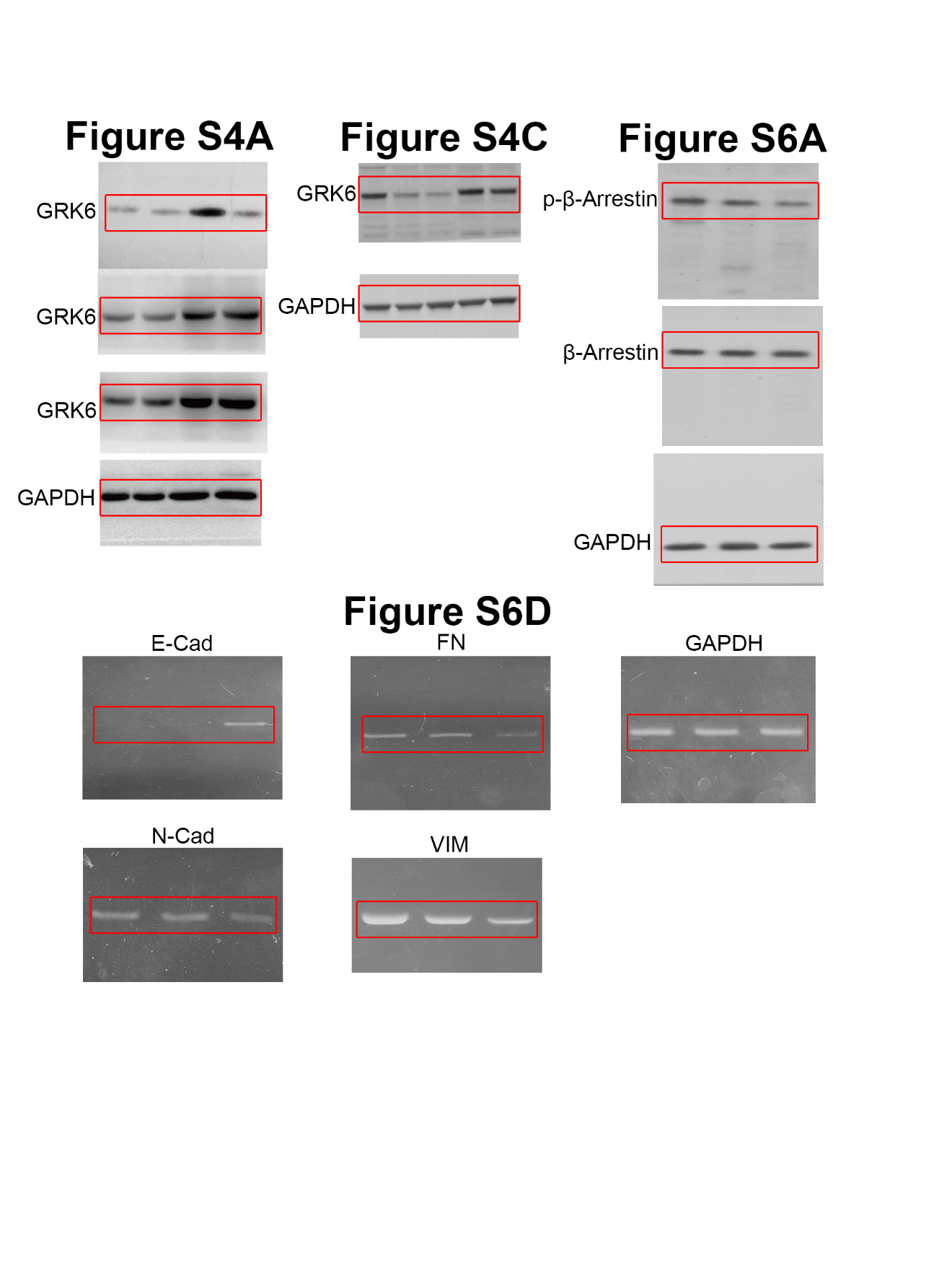
**

**Figure S16. The full uncropped Blots for Figure S4A, S4C, S6A and S6D**
